# Supplementary material for: Challenges in the Highly Selective [3 + 1]-Cycloaddition of an Enoldiazoacetamide to Form a Donor–Acceptor Cis-Cyclobutenecarboxamide
Source: Molecules. 2021 Jun 9;26(12):3520. doi: 10.3390/molecules26123520 (PMC8229219; doi:10.3390/molecules26123520)
Supplement: Supplementary file 1 [file molecules-26-03520-s001.zip › molecules-1242534-SI.pdf]

Supplementary Materials

Challenges in the Highly Selective [3 + 1]-Cycloaddition of an Enoldiazoacetamide to a Donor–Acceptor Cyclopropenecarboxamide

Sipak Joyasawal, Donghui Ma and Michael P. Doyle*

Department of Chemistry, The University of Texas at San Antonio One UTSA Circle, San Antonio, TX 78249, USA; sipak.joyasawal@utsa.edu (S.J.); ma698@purdue.edu (D.M.)

* Correspondence: michael.doyle@UTSA.edu

Table of Contents

1. **Table S1.** Complete Data for Optimization in Catalytic Asymmetric [3 + 1]-Cycloaddition Reactions of **1a** with **2a**/**2b** 2
2. **Table S2.** Solvent screening of [3 + 1]-Cycloaddition Reaction of **1a** with **2b** using ligand **17** 2
3. **Table S3.** Complete Data for Optimization in Catalytic Asymmetric [3 + 1]-Cycloaddition Reaction of donor acceptor cyclopropene **5** with **2b** 3
4. Optimization of Conditions of Diene (**4**) formation 3
5. NMR Spectra 5
6. Selected HPLC Spectra 16

1. **Table S1.** Complete Data for Optimization in Catalytic Asymmetric [3 + 1]-Cycloaddition Reactions of **1a** with **2a**/**2b**.[a]

Entry[a]	Catalyst	Ligand	Ylide	t/h	T/°C	Yield [%][b]	ee [%][c]
1	CuOTf·Tol1/2	**L1**	**2a**	18	rt	58	0
2	CuOTf·Tol1/2	**L1**	**2b**	16	rt	85	−17
3	CuOTf·Tol1/2	**L2**	**2b**	24	0	84	−34
4	CuOTf·Tol1/2	**L3**	**2b**	24	0	87	34
5	CuOTf·Tol1/2	**L3**	**2a**	24	rt	57	−5
6	CuOTf·Tol1/2	**L5**	**2b**	24	rt	74	−64
7	CuOTf·Tol1/2	**L6**	**2b**	16	rt	60	10
8	CuOTf·Tol1/2	**L7-RS**	**2b**	16	rt	79	14
9	CuOTf·Tol1/2	**L8-SR**	**2b**	16	rt	83	−14
10	CuOTf·Tol1/2	**L9**	**2b**	16	rt	71	32
11	CuOTf·Tol1/2	**L10**	**2b**	16	rt	76	45
12	CuOTf·Tol1/2	**L12**	**2a**	24	rt	60	5
13	CuOTf·Tol1/2	**L12**	**2b**	16	rt	90	38
14	CuOTf·Tol1/2	**L13**	**2b**	16	rt	78	44
15	CuOTf·Tol1/2	**L14**	**2b**	16	rt	54	34
16	CuOTf·Tol1/2	**L14**	**2b**	24	0	60	38
17	CuOTf·Tol1/2	**L16**	**2b**	24	rt	80	58
18	CuOTf·Tol1/2	**L17**	**2a**	24	rt	55	5
19	CuOTf·Tol1/2	**L17**	**2b**	16	rt	79	60
20	CuOTf·Tol1/2	**L17**	**2b**	24	0	82	64
21[d]	CuOTf·Tol1/2	**L17**	**2b**	24	−20	74	68
22	$CuBF_4(CH_3CN)_4$	**L17**	**2b**	16	rt	79	59
23	$CuBF_4(CH_3CN)_4$	**L17**	**2b**	16	0	75	65
24[d]	$CuBF_4(CH_3CN)_4$	**L17**	**2b**	24	−20	82	67

[a] Unless otherwise noted, the reactions were performed as follows: to a solution of CuOTf·Tol1/2 (0.01 mmol, 5 mol%), and ligand (0.012 mmol, 6 mol%) in dry DCM (1.0 mL) under N_2 was stirred at rt for 1 h, after which sulfur ylide **2a**/or **2b** (0.20 mmol) in dry CH_2Cl_2 (0.5 mL) was added **1a** (0.24 mmol) in dry DCM (0.5 mL at the specified temperature. The reaction solution was stirred at the same temperature for the indicated time. [b] Isolated yield after chromatography. [c] Enantiomeric excess was determined by HPLC analysis on a Chiralpak OD-H column. Negative values specify the opposite enantiomer. [d] All reactions at –20 °C were performed in a freezer.

2. **Table S2.** Solvent screening of [3 + 1]-Cycloaddition Reaction of **1a** with **2b** using ligand **17**:[a]

Entry[a]	Ligand	Solvent	T/°C	t/h	Yield[b]	ee[d]
1	**L17**	DCE	rt	18	33%	48%
2	**L17**	$CHCl_3$	rt	36	0	0
3	**L17**	THF	rt	18	66%	42%
4	**L17**	Et_2O	rt	36	26%	20%
5	**L17**	Toluene	rt	36	20%	−14%

[a] Unless otherwise noted, the reactions were performed as follows: a solution of CuOTf·Tol1/2 (0.01 mmol, 5 mol%), and ligand (0.012 mmol, 6 mol%) in dry solvent (1.0 mL) under N_2 was stirred at rt for 1 h, after which sulfur ylide **2b** (0.20 mmol) in dry solvent (0.5 mL) was added followed by dropwise addition of cyclopropenecarboxamide **1a** (0.24 mmol) in dry solvent (0.5 mL) at the specific temperature. The reaction

solution was stirred at the same temperature for the indicated time. [b] Isolated yield after chromatography. [c]Enantiomeric excess was determined by HPLC analysis on a Chiralpak OD-H column.

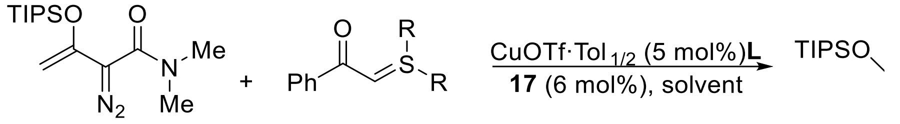

1a **2b**. R = Ph

Scheme S1. Solvent screening of [3 + 1]-cycloaddition reaction of **1a** with **2b** using ligand **17.**

3. Table S3. Complete Data for Optimization in Catalytic Asymmetric [3 + 1]-Cycloaddition Reaction of Donor-acceptor Cyclopropene **5** with **2b**.[a]

Entry[a]	ligand	solvent	T/°C	t/h	dr (*Z*:*E*)[b]	Yield (ee) *Z*-3b[c,e]	Yield (ee) *E*-3b[d,e]	Yield (4)[c]
1[g]	**L17**	DCM	rt	24	(7:1)	35% (54%)	5% (4%)	52%
2[g]	**L14**	DCM	rt	24	(6:1)	57% (64%)	10% (7%)	22%
3[h]	**L14**	DCM	rt	24	(9:1)	69% (68%)	8% (20%)	15%
4	**L1**	DCM	rt	38	(2:1)	32% (−12%)	12% (65%)	47%
5	**L4**	DCM	rt	24	(2.5:1)	50% (0%)	18% (0%)	4%
6	**L5**	DCM	rt	24	(8:1)	28% (−69%)	3% (62%)	59%
7	**L11**	DCM	rt	24	(5:1)	40% (62%)	8% (56%)	44%
8	**L12**	DCM	rt	24	(5:1)	61% (48%)	12% (10%)	16%
9	**L14**	DCM	rt	24	(12:1)	67% (71%)	5.5% (16%)	8%
10[f]	**L14**	DCM	24	48	(>20:1)	73% (79%)	3% (32%)	2%
11	**L16**	DCM	rt	38	(10:1)	57% (59%)	6% (NA)	22%
12	**L17**	DCM	rt	24	(18:1)	24% (63%)	2% (NA)	51%

[a] Unless otherwise noted, reactions were performed as follows: a solution of CuOTf·Tol$_{1/2}$ (0.01 mmol, 5 mol%), and ligand **L** (0.012 mmol, 6 mol%) in dry CH_2Cl_2 (1.0 mL) under N_2 was stirred at rt for 1 h, after which sulfur ylide **2b** (0.20 mmol) in dry CH_2Cl_2 (0.5 mL) was added and then cyclopropene **5** (0.24 mmol) in dry CH_2Cl_2 (0.5 mL) was added dropwise at the specific temperature; the reaction solution was stirred at the same temperature for the indicated time. [b] dr calculated from the reaction mixture after completion of the reaction; [c] Isolated yield of the ***Z*** diastereomer and diene (**4**); [d] Yield of the ***E*** diastereomer was calculated from the ^{1}H-NMR analysis of the reaction mixture; [e] Enantiomeric excess was determined by HPLC analysis on a Chiralpak OD-H column. [f] Reaction at −20 °C was performed in a freezer, [g] Starting material is **1b** (***Z*:*E*** = 1:3); [h] Starting material is **1b** (***Z*:*E*** = 25:1); NA = Not available.

4. Optimization of Conditions of Diene (**4**) formation**:**

When **1b** (***Z*:*E*** = 25:1) or **5** (Scheme S2) was treated individually with **L17**, **2b,** or CuOTf·Tol$_{1/2}$ under the standard conditions none of them showed even a trace of diene **4**. However, when **1b** (***Z*:*E*** = 25:1) or **5** (Scheme S3) was treated with CuOTf·Tol$_{1/2}$ in the presence of ligand (**L14** or **L17**) or Et_3N, formation of dine **4** (~30%) was observed in the reported amounts.

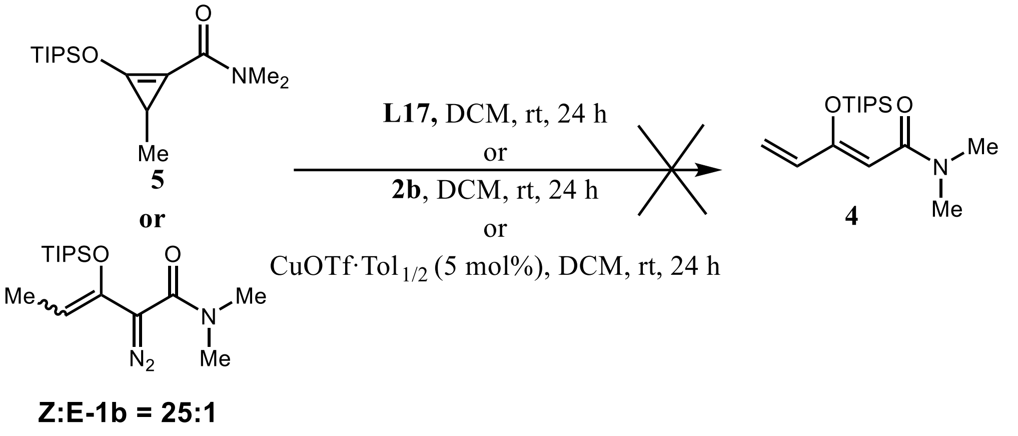

Scheme S2. Reactants not involved in diene (**4**) formation.

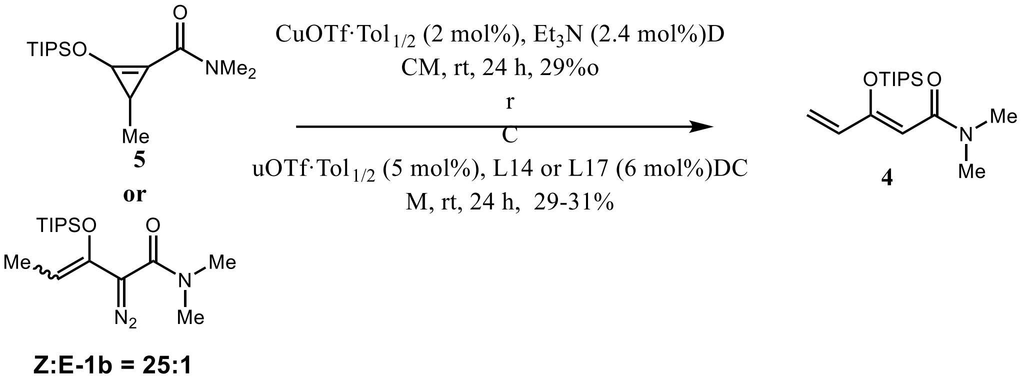

Scheme S3. Optimization of conditions of diene (**4**) formation.

5. NMR Spectra:

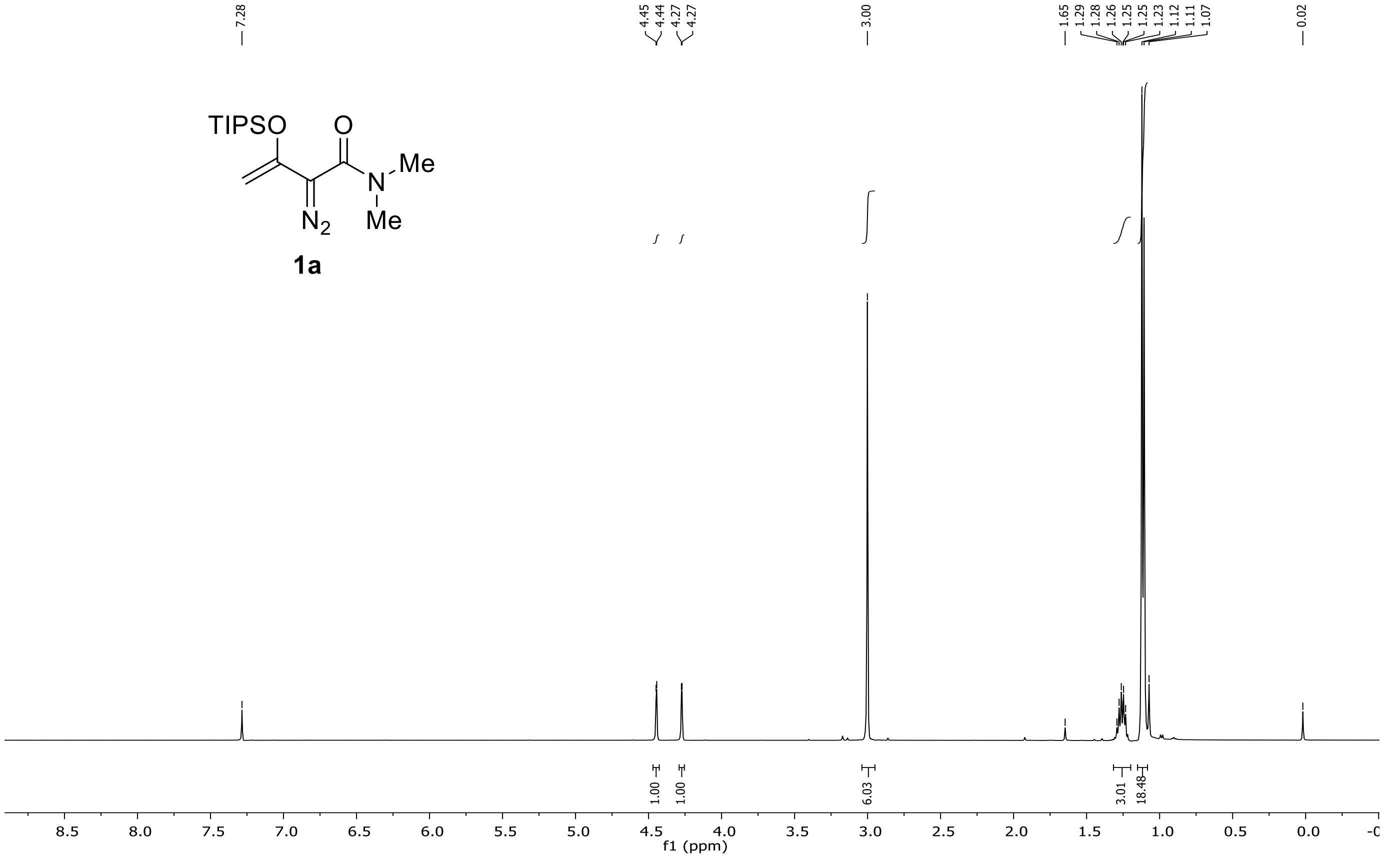

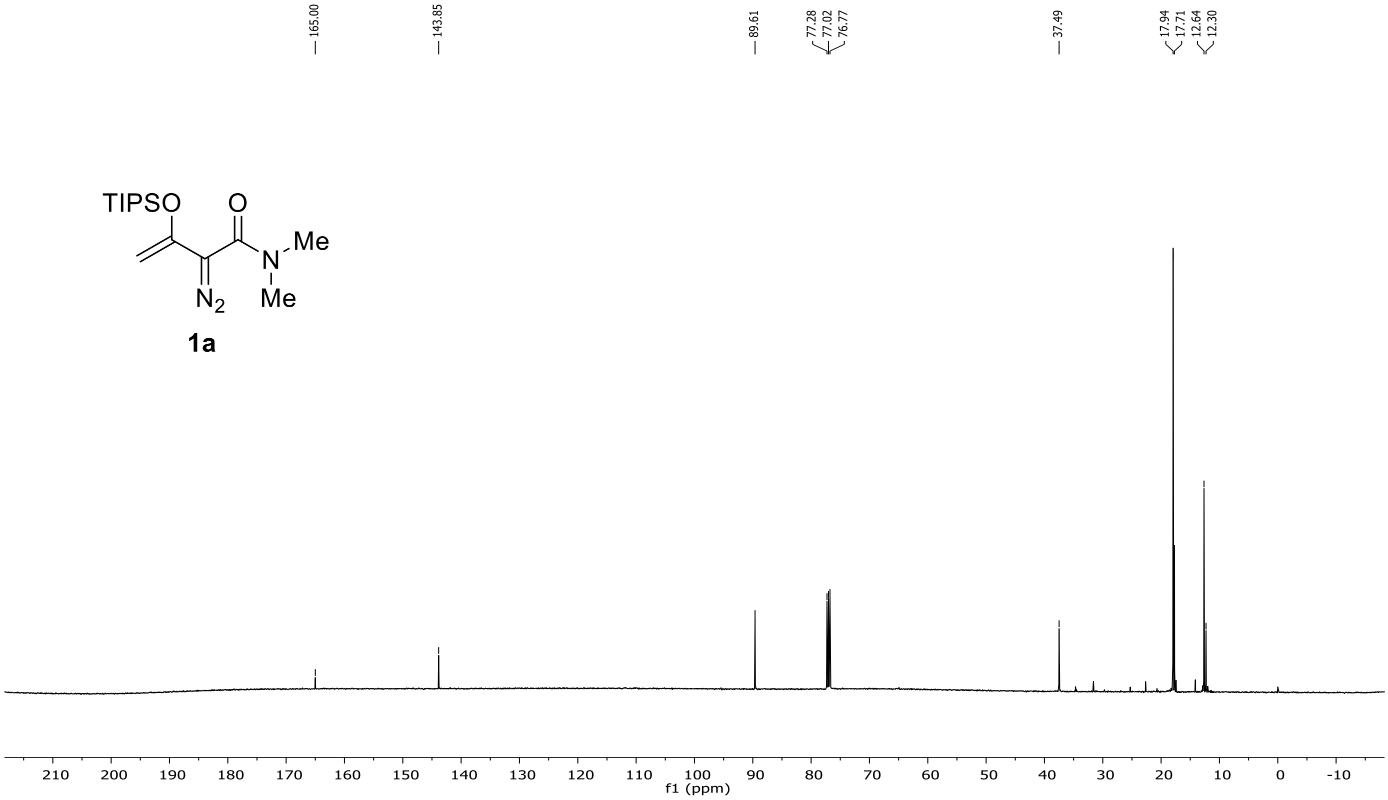

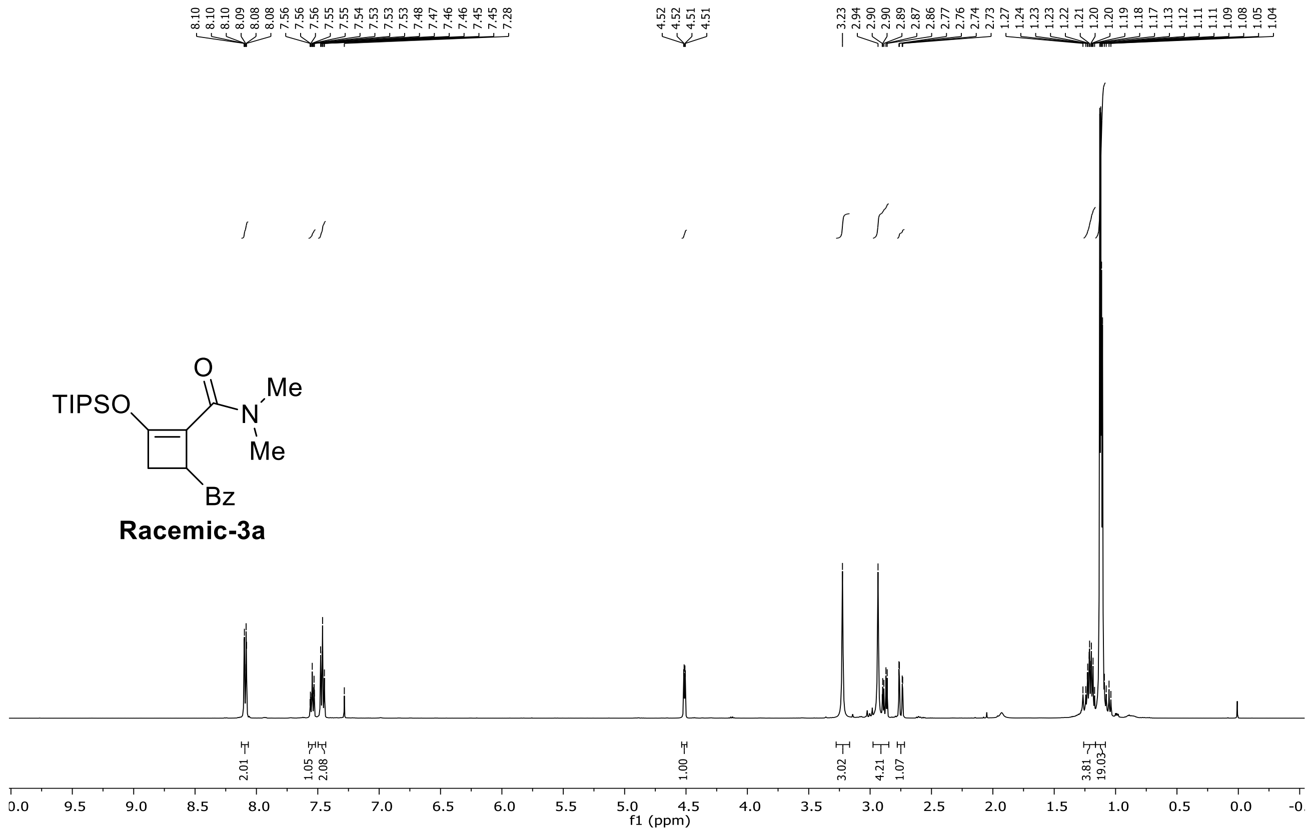
8.10 8.10 8.10 8.09 8.08 8.08 7.56 7.56 7.55 7.55 7.54 7.53 7.53 7.53 7.48 7.47 7.46 7.46 7.45 7.45 7.28
4.52 4.52 4.51 4.51
3.23 2.94 2.90 2.90 2.89 2.87 2.86 2.77 2.76 2.74 2.73 1.27 1.24 1.23 1.23 1.22 1.21 1.20 1.20 1.19 1.18 1.17 1.13 1.12 1.11 1.11 1.09 1.08 1.05 1.04
TIPSO
O
N
Me
Me
Bz
Racemic-3a
2.01
1.05
2.08
1.00
3.02
4.21
1.07
3.81
19.03
f1 (ppm)

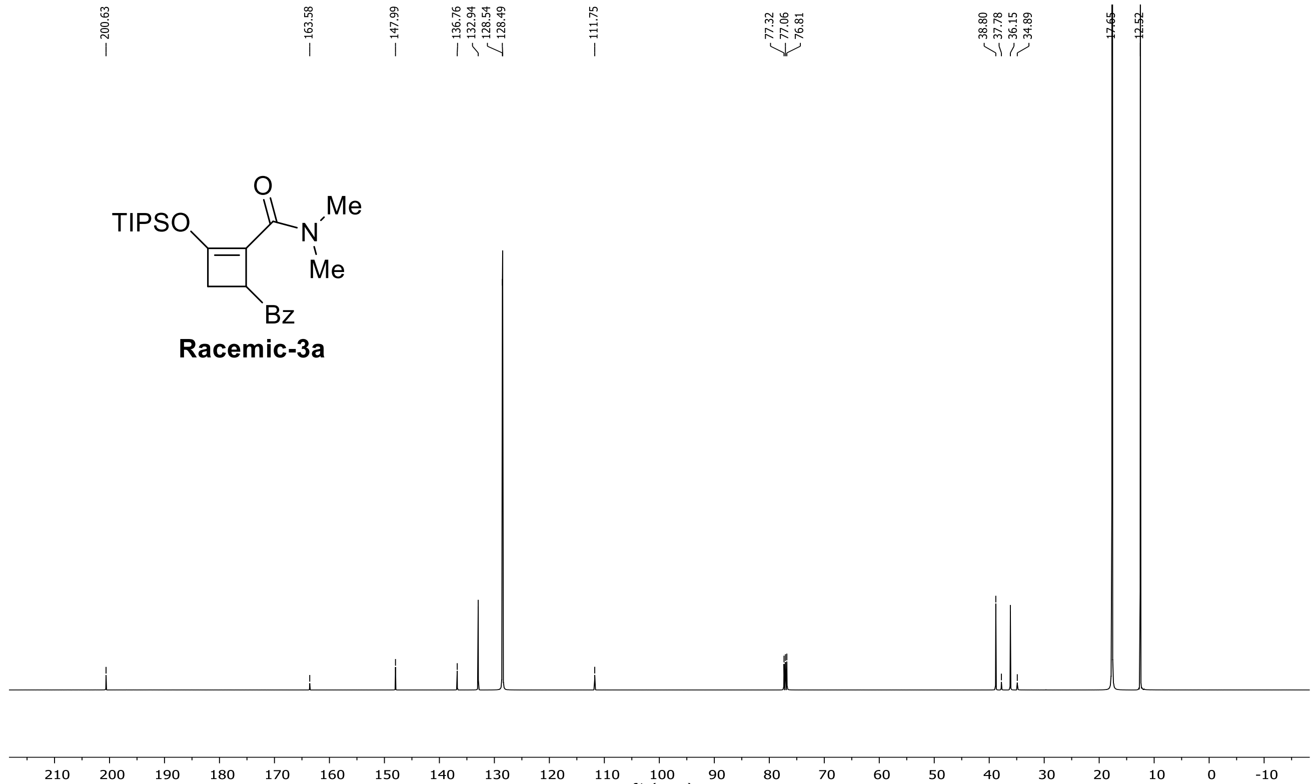
200.63
163.58
147.99
136.76 132.94 128.54 128.49
111.75
77.32 77.06 76.81
38.80 37.78 36.15 34.89
17.65
12.52
TIPSO
O
N
Me
Me
Bz
Racemic-3a
f1 (ppm)

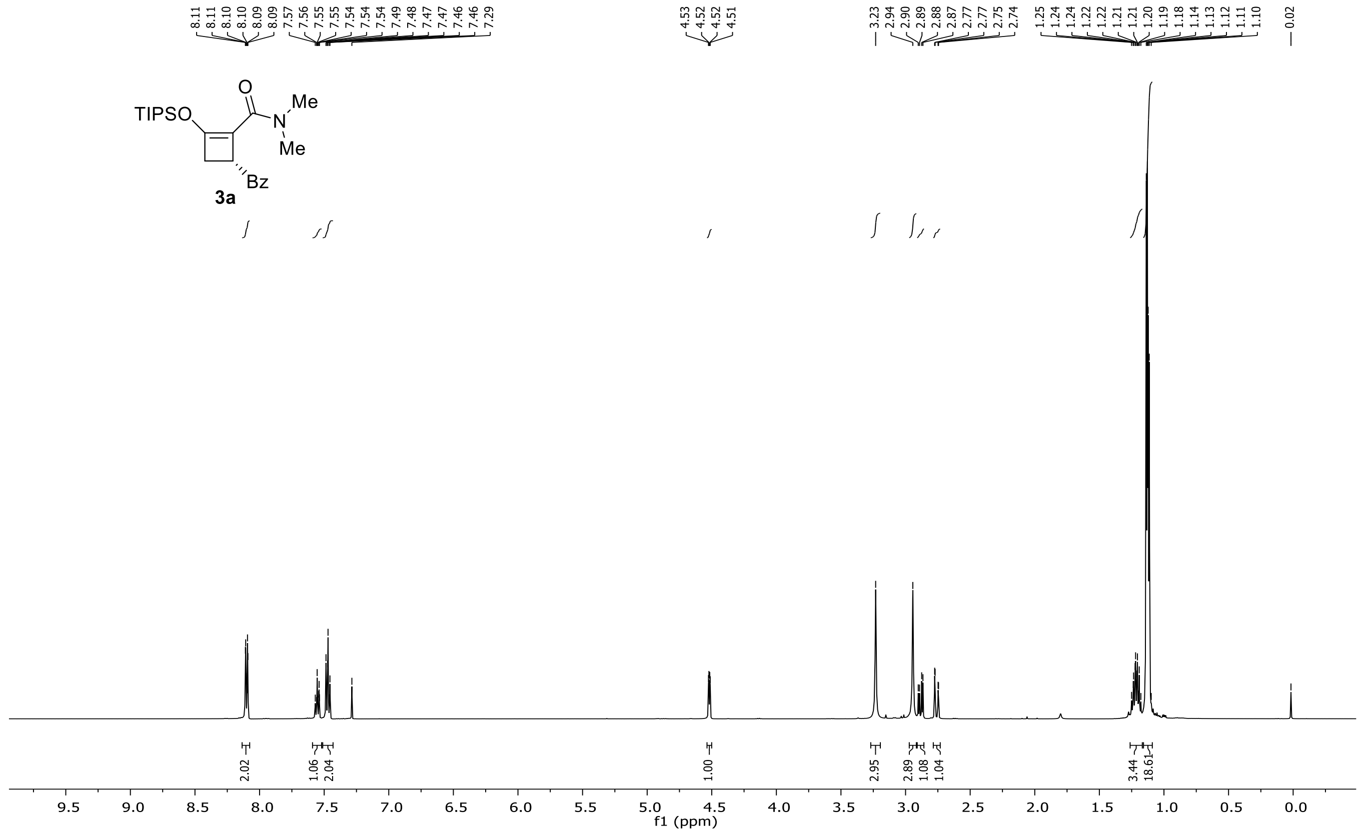

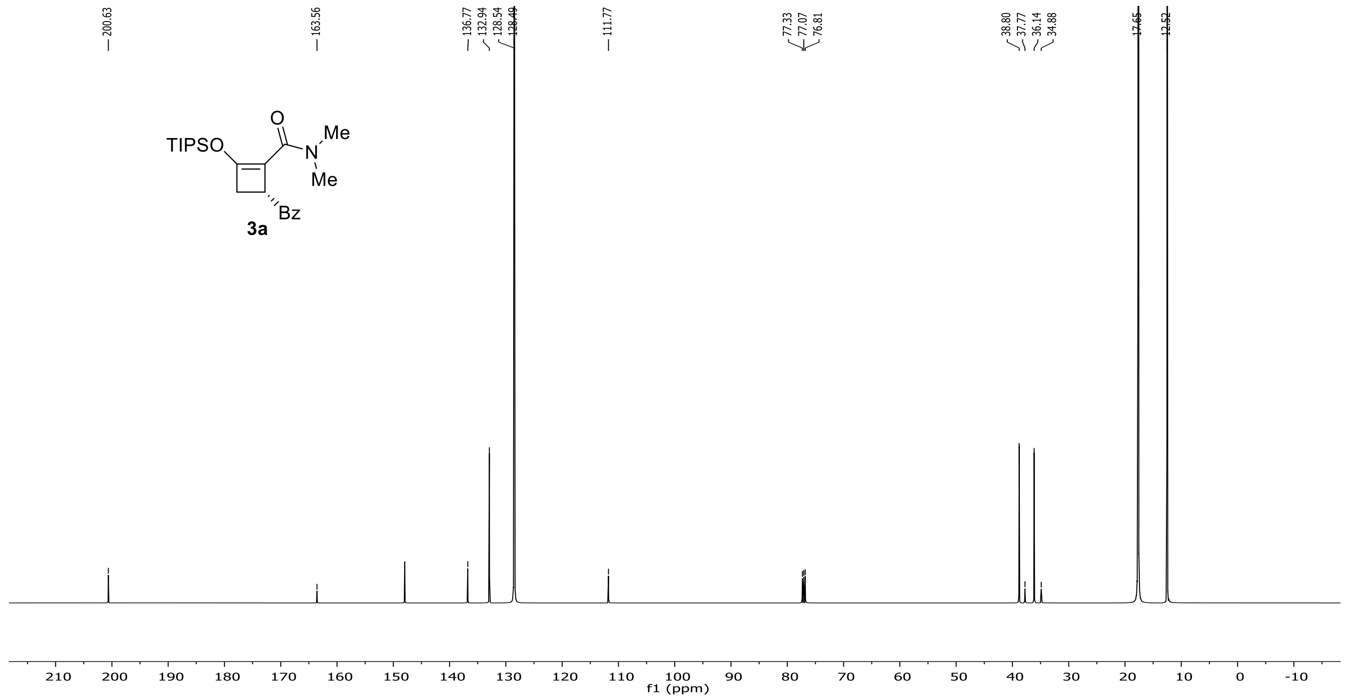

7.NMR Spectra of Cyclobutenecarboxamides **Z-3b** and **4**

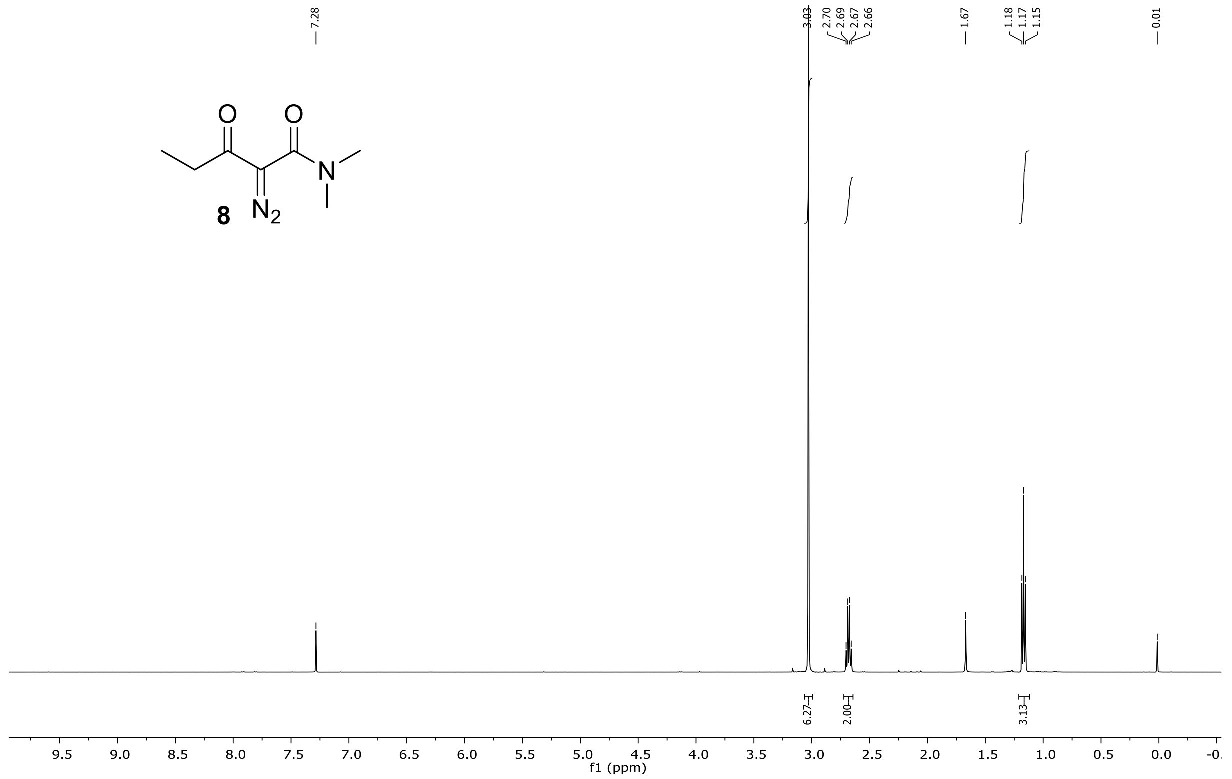

7.28
3.03
2.70
2.69
2.67
2.66
1.67
1.18
1.17
1.15
0.01
O
O
N
8
N2
6.27
2.00
3.13
f1 (ppm)

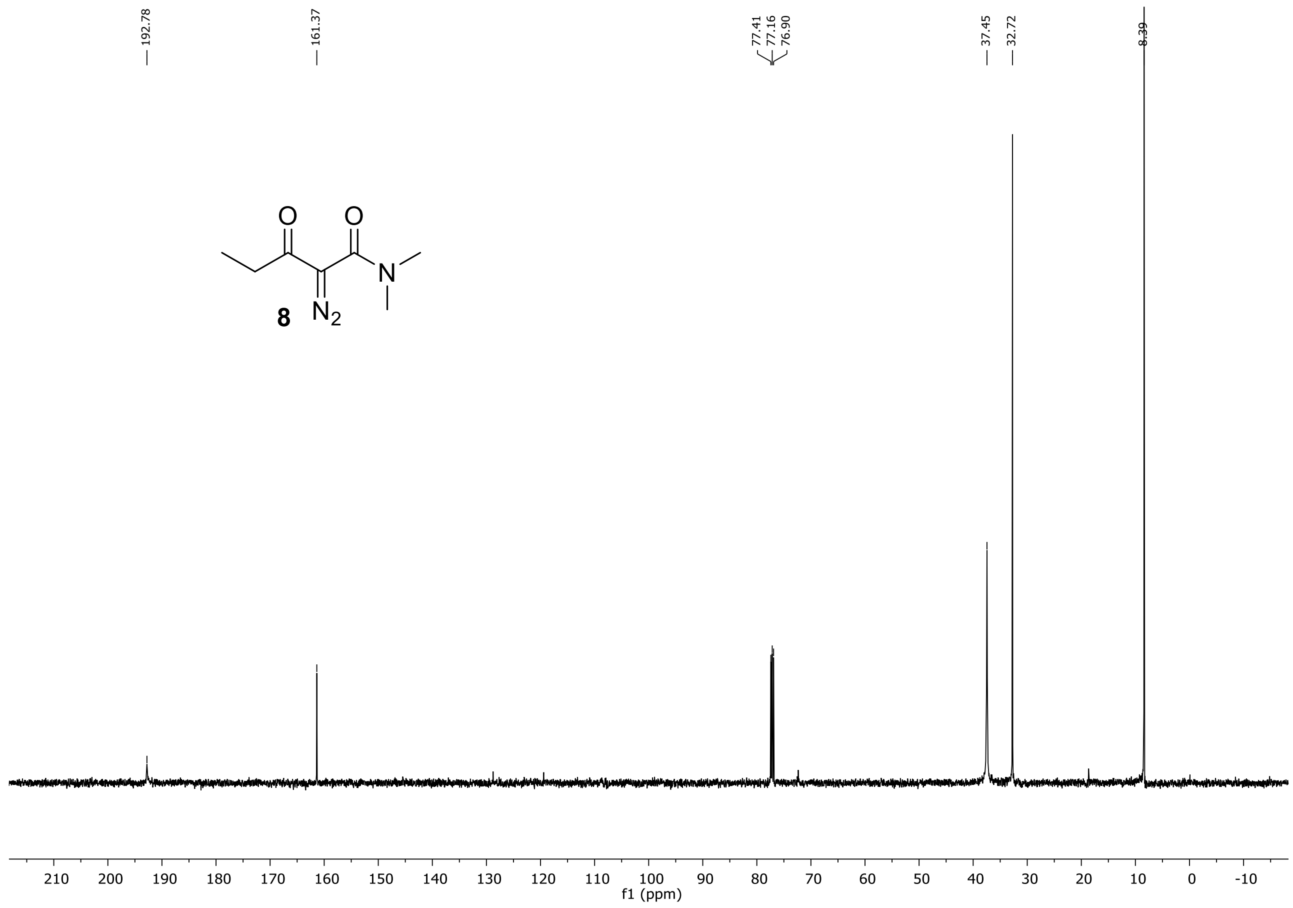

192.78
161.37
77.41
77.16
76.90
37.45
32.72
8.39
O
O
N
8
N2
f1 (ppm)

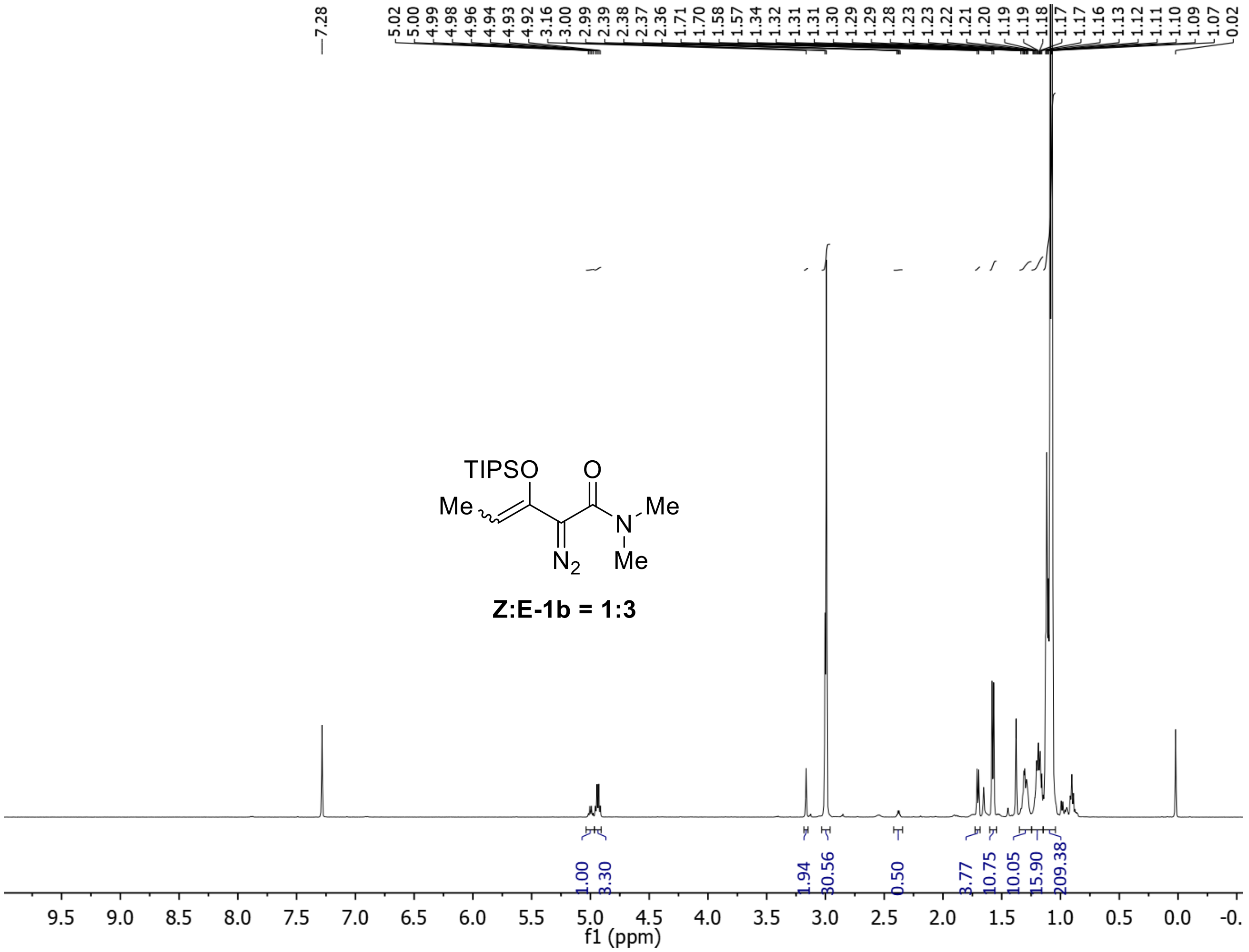
TIPSO
O
Me
N
Me
N_2
Me
Z:E-1b = 1:3
—7.28
5.02 5.00 4.99 4.98 4.96 4.94 4.93 4.92 3.16 3.00 2.99 2.39 2.38 2.37 2.36 1.71 1.70 1.58 1.57 1.34 1.32 1.31 1.31 1.30 1.29 1.29 1.28 1.23 1.23 1.22 1.21 1.20 1.19 1.19 1.18 1.17 1.16 1.13 1.12 1.11 1.10 1.09 1.07 0.02
1.00 3.30 1.94 30.56 0.50 3.77 10.75 10.05 15.90 209.38
9.5 9.0 8.5 8.0 7.5 7.0 6.5 6.0 5.5 5.0 4.5 4.0 3.5 3.0 2.5 2.0 1.5 1.0 0.5 0.0
f1 (ppm)

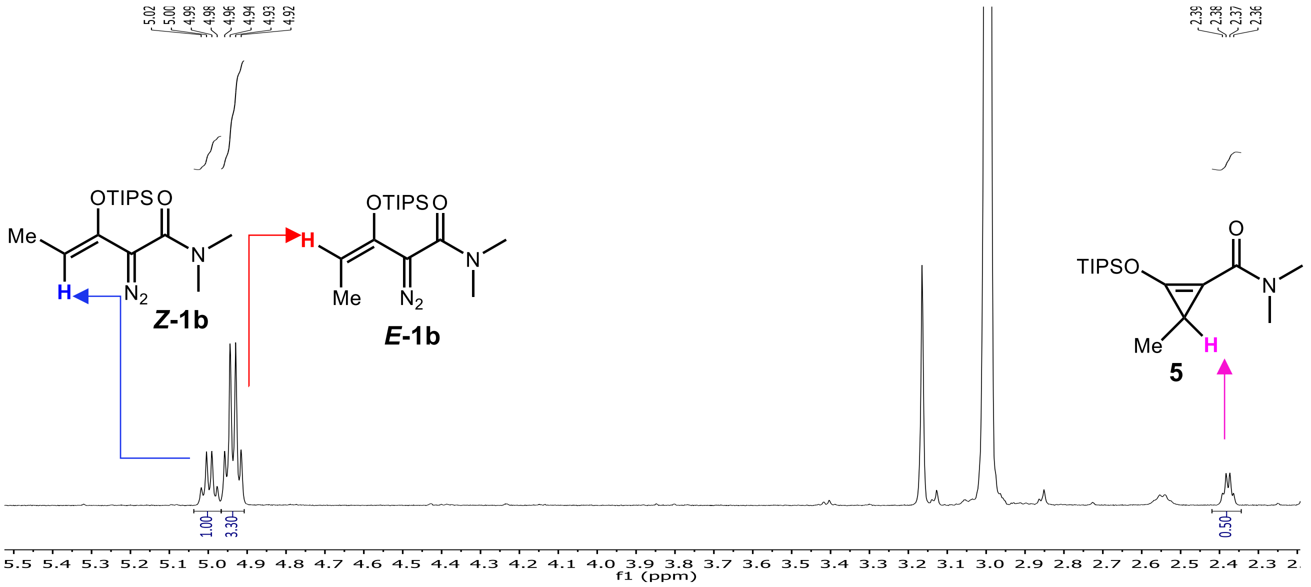
OTIPS O
Me
N
H
N_2
Z-1b
OTIPS O
H
N
Me
N_2
E-1b
TIPSO
O
N
Me
H
5
5.02 5.00 4.99 4.98 4.96 4.94 4.93 4.92
2.39 2.38 2.37 2.36
1.00 3.30
0.50
5.5 5.4 5.3 5.2 5.1 5.0 4.9 4.8 4.7 4.6 4.5 4.4 4.3 4.2 4.1 4.0 3.9 3.8 3.7 3.6 3.5 3.4 3.3 3.2 3.1 3.0 2.9 2.8 2.7 2.6 2.5 2.4 2.3
f1 (ppm)

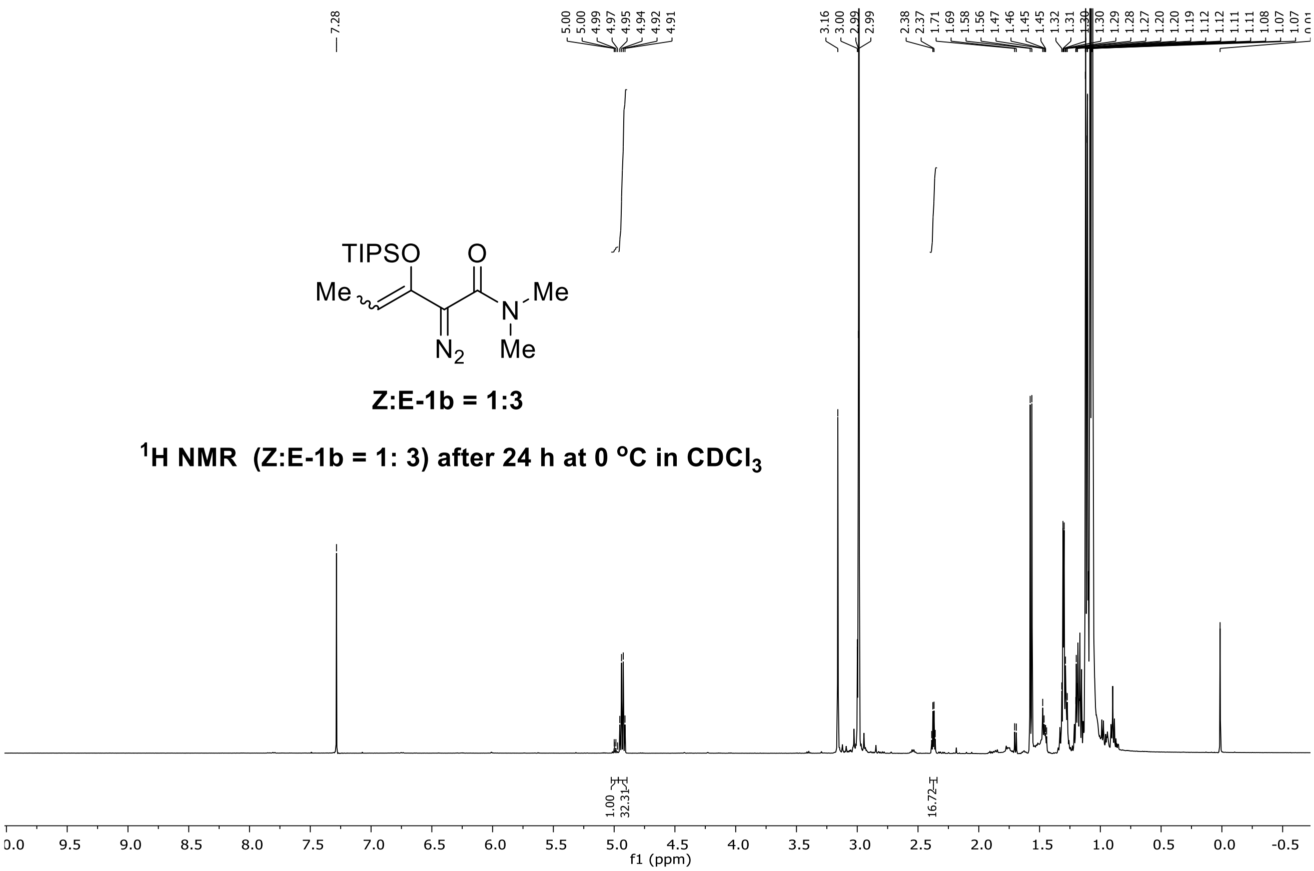
TIPSO
O
Me
N
Me
N2
Me
Z:E-1b = 1:3
1H NMR (Z:E-1b = 1: 3) after 24 h at 0 oC in CDCl3
f1 (ppm)

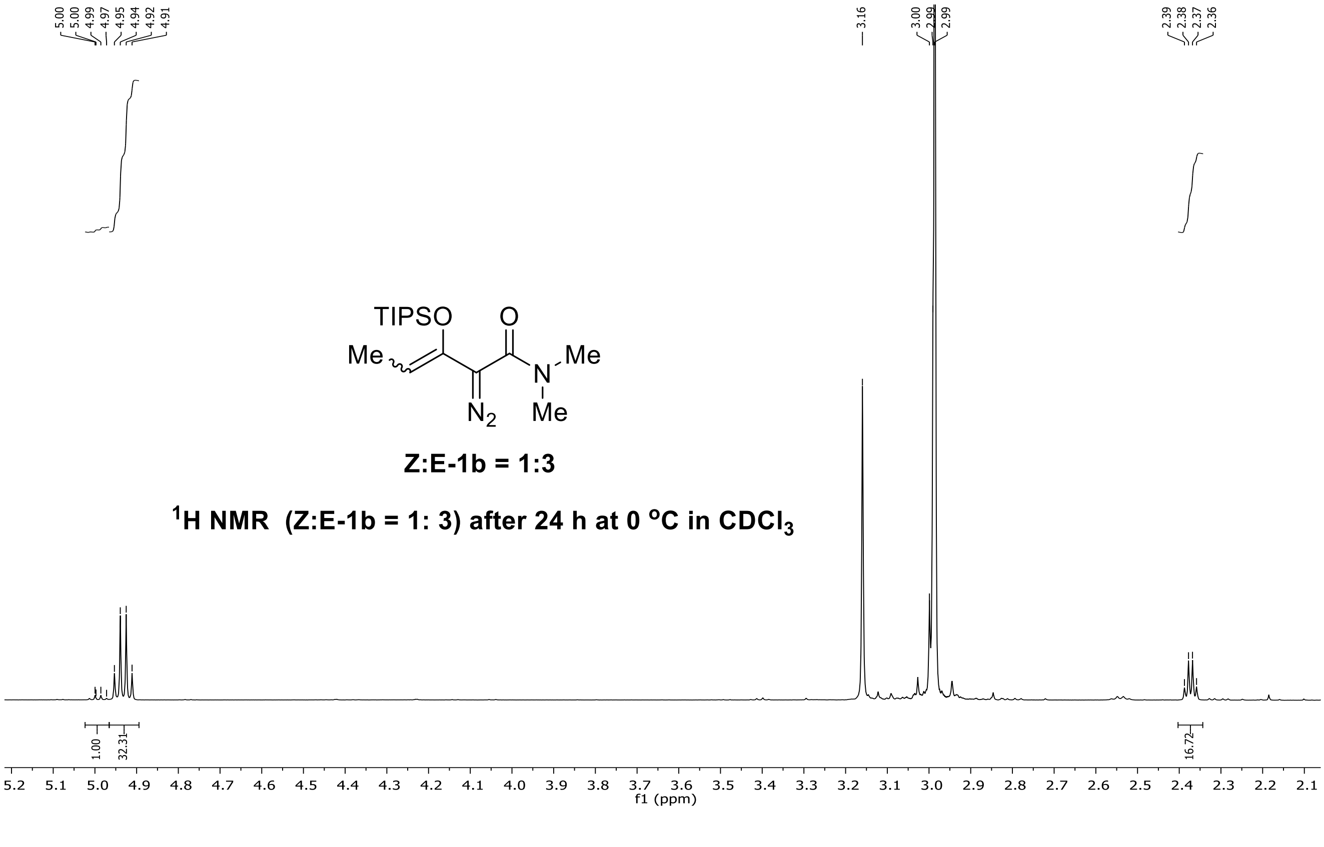
TIPSO
O
Me
N
Me
N2
Me
Z:E-1b = 1:3
1H NMR (Z:E-1b = 1: 3) after 24 h at 0 oC in CDCl3
f1 (ppm)

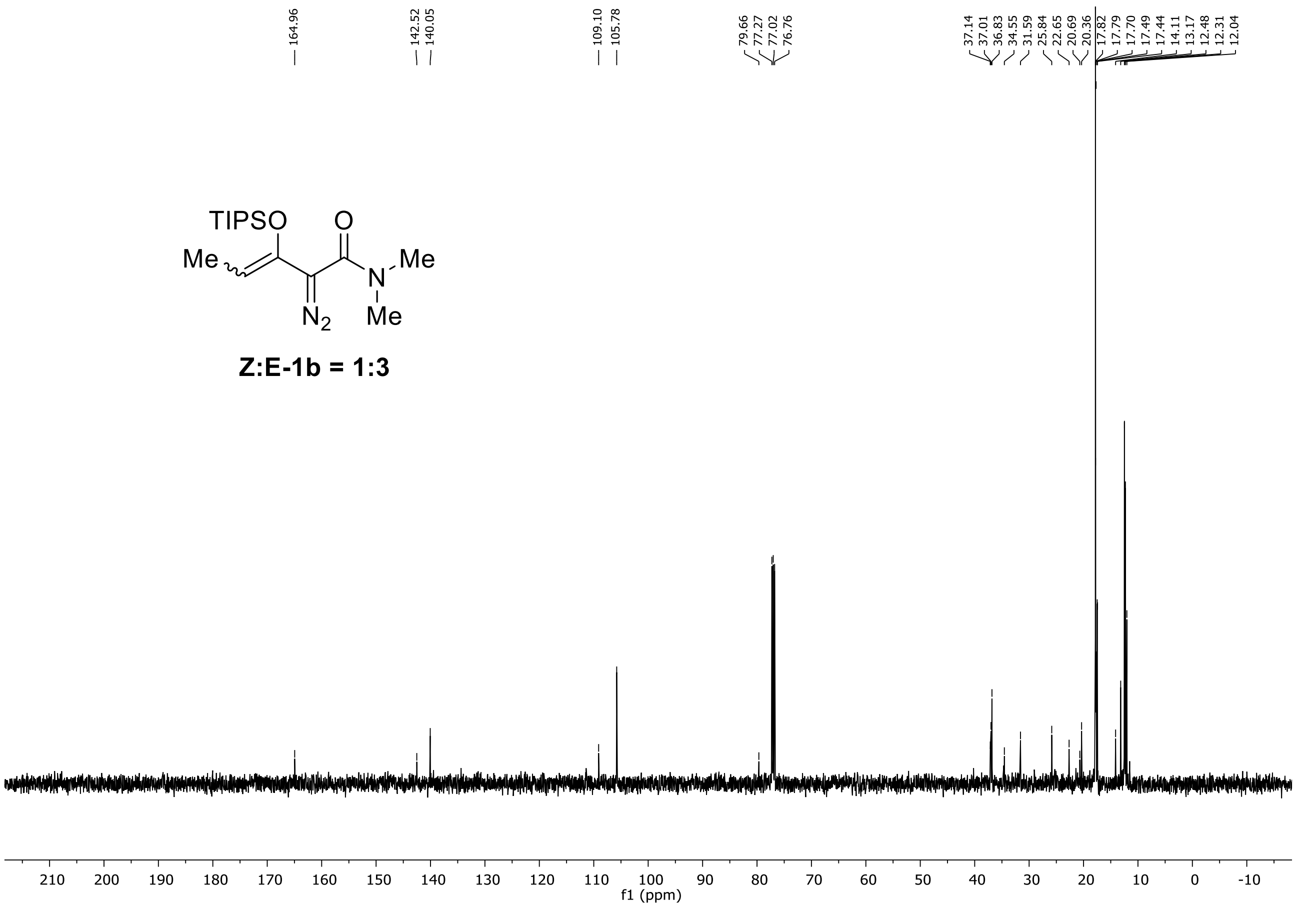
164.96
142.52
140.05
109.10
105.78
79.66
77.27
77.02
76.76
37.14
37.01
36.83
34.55
31.59
25.84
22.65
20.69
20.36
17.82
17.79
17.70
17.49
17.44
14.11
13.17
12.48
12.31
12.04
TIPSO
O
Me
N
Me
N2
Me
Z:E-1b = 1:3
210 200 190 180 170 160 150 140 130 120 110 100 90 80 70 60 50 40 30 20 10 0 -10
f1 (ppm)

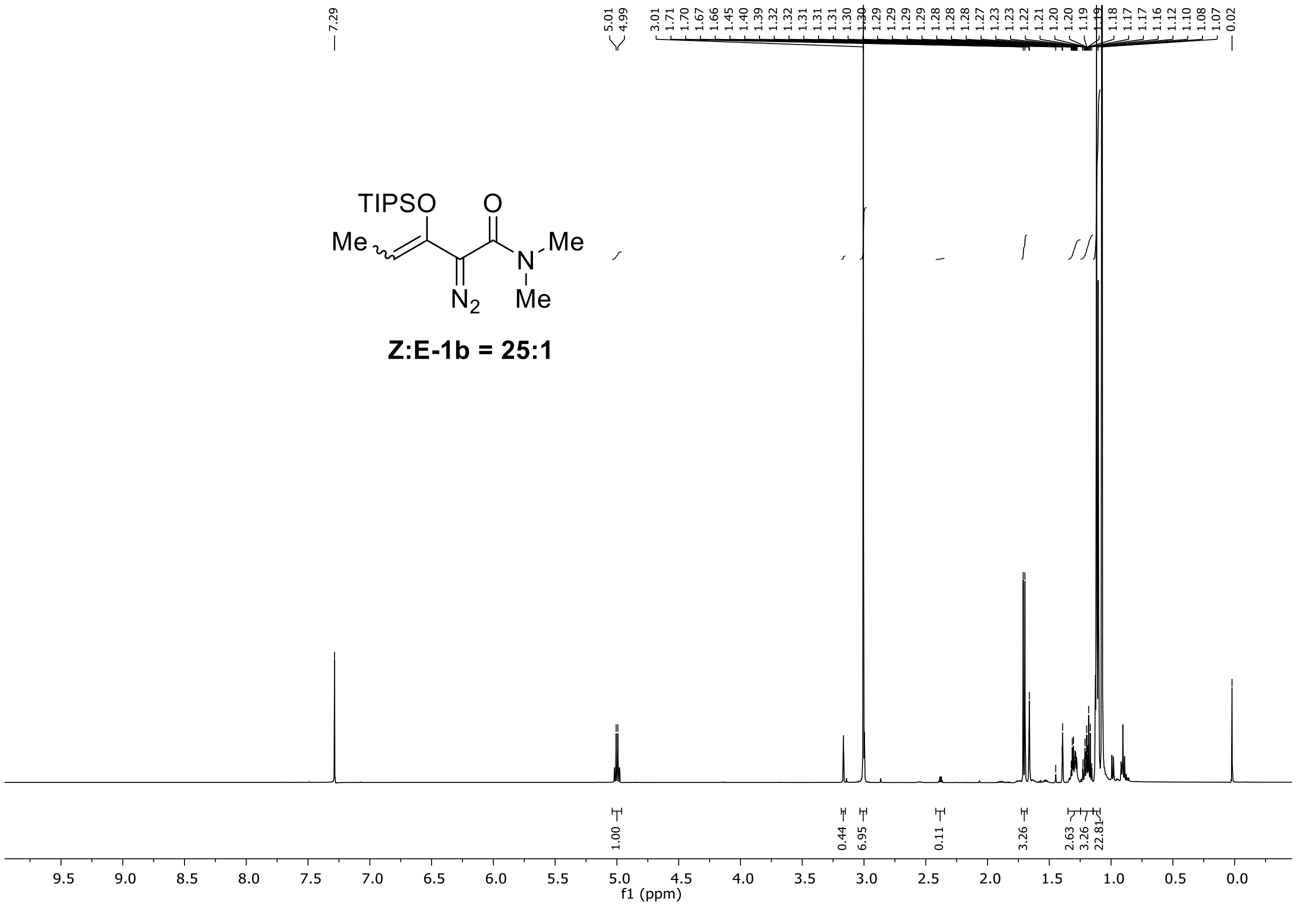
7.29
5.01
4.99
3.01
1.71
1.70
1.67
1.66
1.45
1.40
1.39
1.32
1.32
1.31
1.31
1.30
1.30
1.29
1.29
1.29
1.29
1.28
1.28
1.28
1.27
1.23
1.23
1.22
1.21
1.20
1.19
1.19
1.18
1.18
1.17
1.17
1.16
1.12
1.10
1.08
1.07
0.02
TIPSO
O
Me
N
Me
N2
Me
Z:E-1b = 25:1
1.00
0.44
6.95
0.11
3.26
2.63
3.26
22.81
9.5 9.0 8.5 8.0 7.5 7.0 6.5 6.0 5.5 5.0 4.5 4.0 3.5 3.0 2.5 2.0 1.5 1.0 0.5 0.0
f1 (ppm)

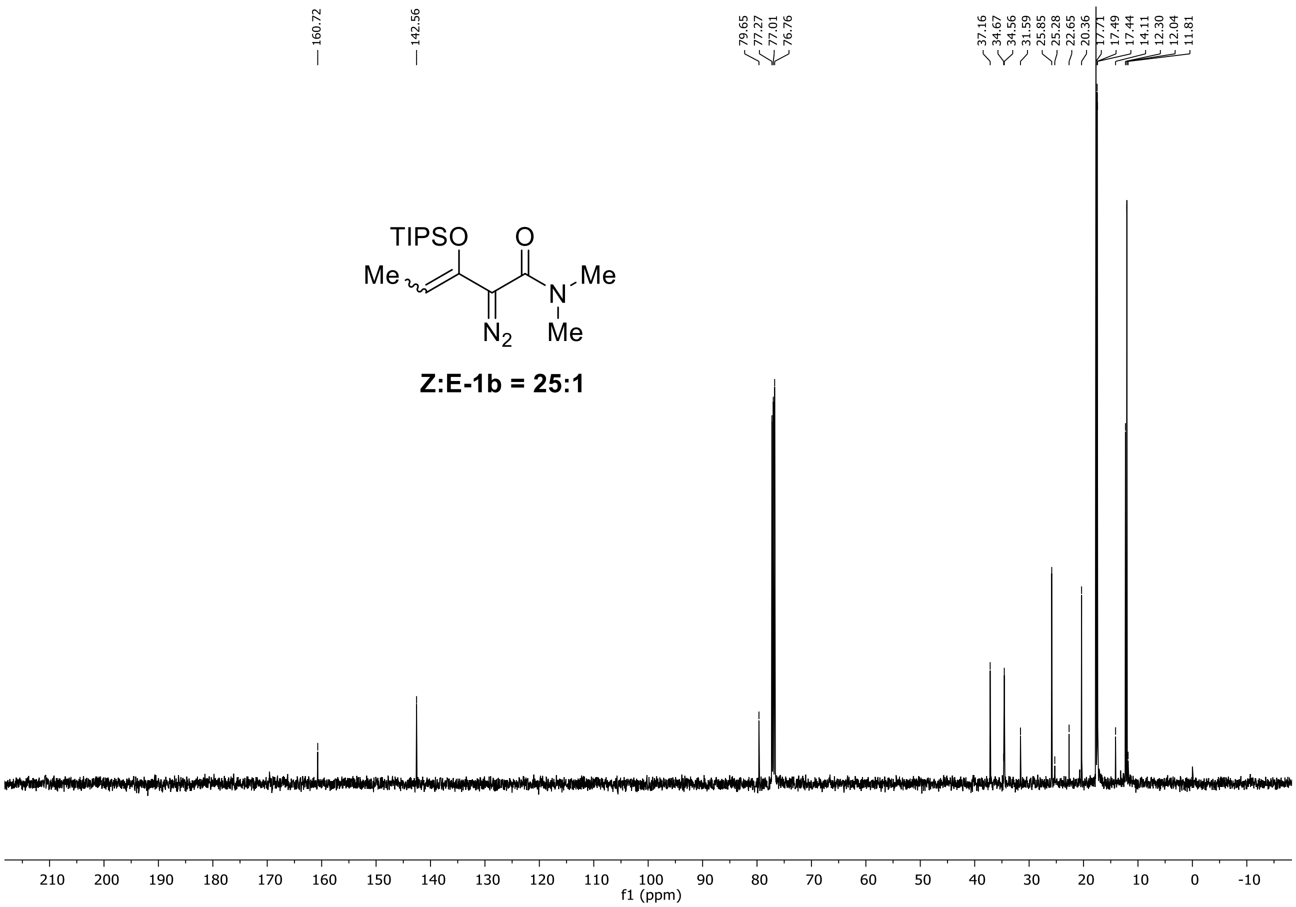
160.72
142.56
79.65
77.27
77.01
76.76
37.16
34.67
34.56
31.59
25.85
25.28
22.65
20.36
17.71
17.49
17.44
14.11
12.30
12.04
11.81
TIPSO
O
Me
N
Me
N2
Me
Z:E-1b = 25:1
f1 (ppm)

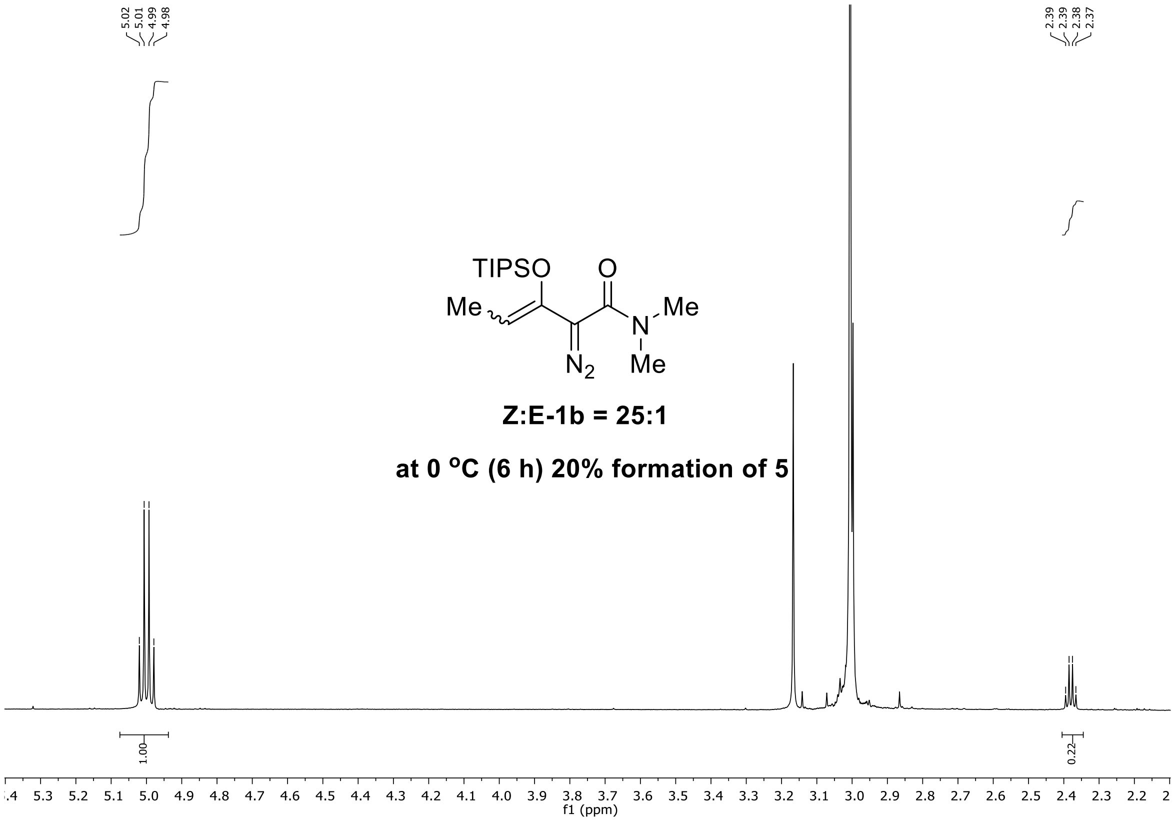
5.02
5.01
4.99
4.98
2.39
2.39
2.38
2.37
TIPSO
O
Me
N
Me
N2
Me
Z:E-1b = 25:1
at 0 °C (6 h) 20% formation of 5
1.00
0.22
f1 (ppm)

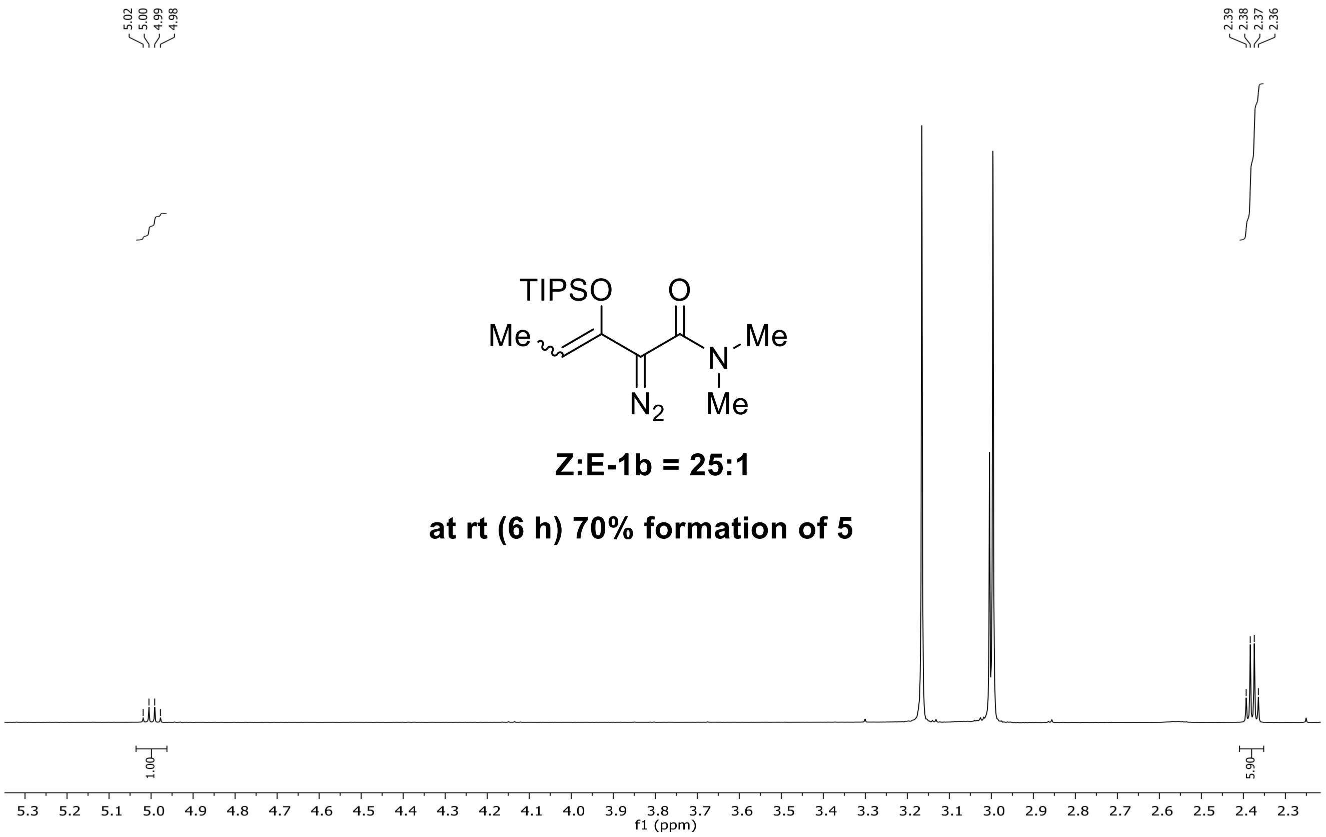
5.02
5.00
4.99
4.98
2.39
2.38
2.37
2.36
TIPSO
O
Me
N
Me
N2
Me
Z:E-1b = 25:1
at rt (6 h) 70% formation of 5
1.00
5.90
f1 (ppm)

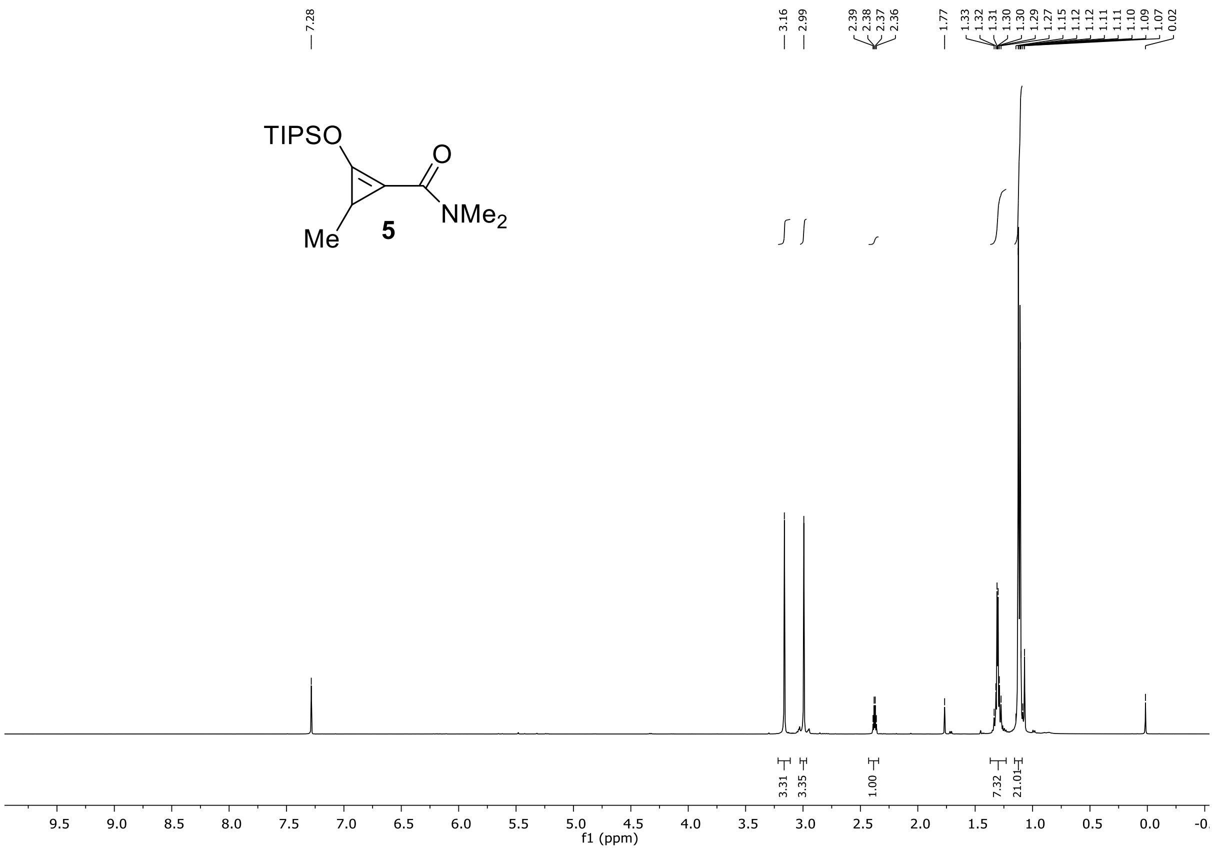
7.28
3.16
2.99
2.39
2.38
2.37
2.36
1.77
1.33
1.32
1.31
1.30
1.30
1.29
1.27
1.15
1.12
1.12
1.11
1.11
1.10
1.09
1.07
0.02
TIPSO
O
NMe2
Me
5
3.31
3.35
1.00
7.32
21.01
f1 (ppm)

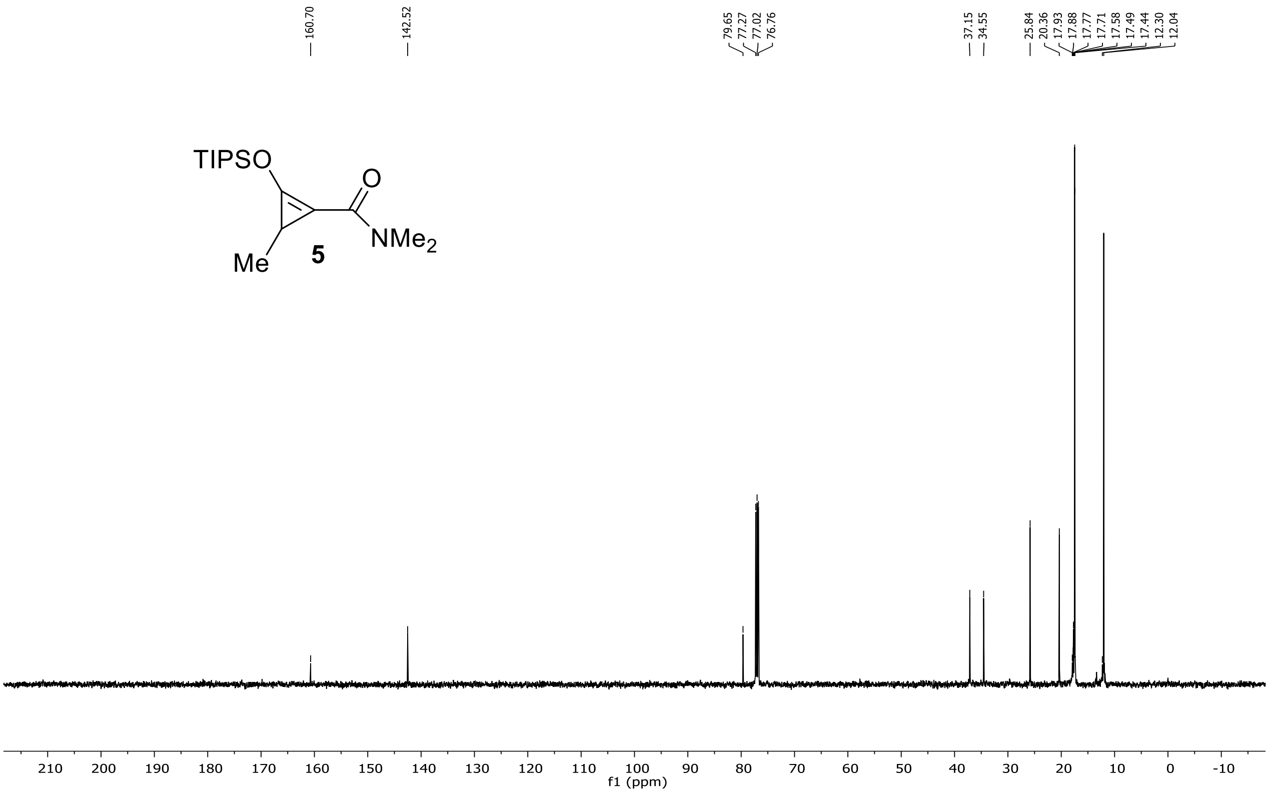
160.70
142.52
79.65
77.27
77.02
76.76
37.15
34.55
25.84
20.36
17.93
17.88
17.77
17.71
17.58
17.49
17.44
12.30
12.04
TIPSO
O
NMe2
Me
5
f1 (ppm)

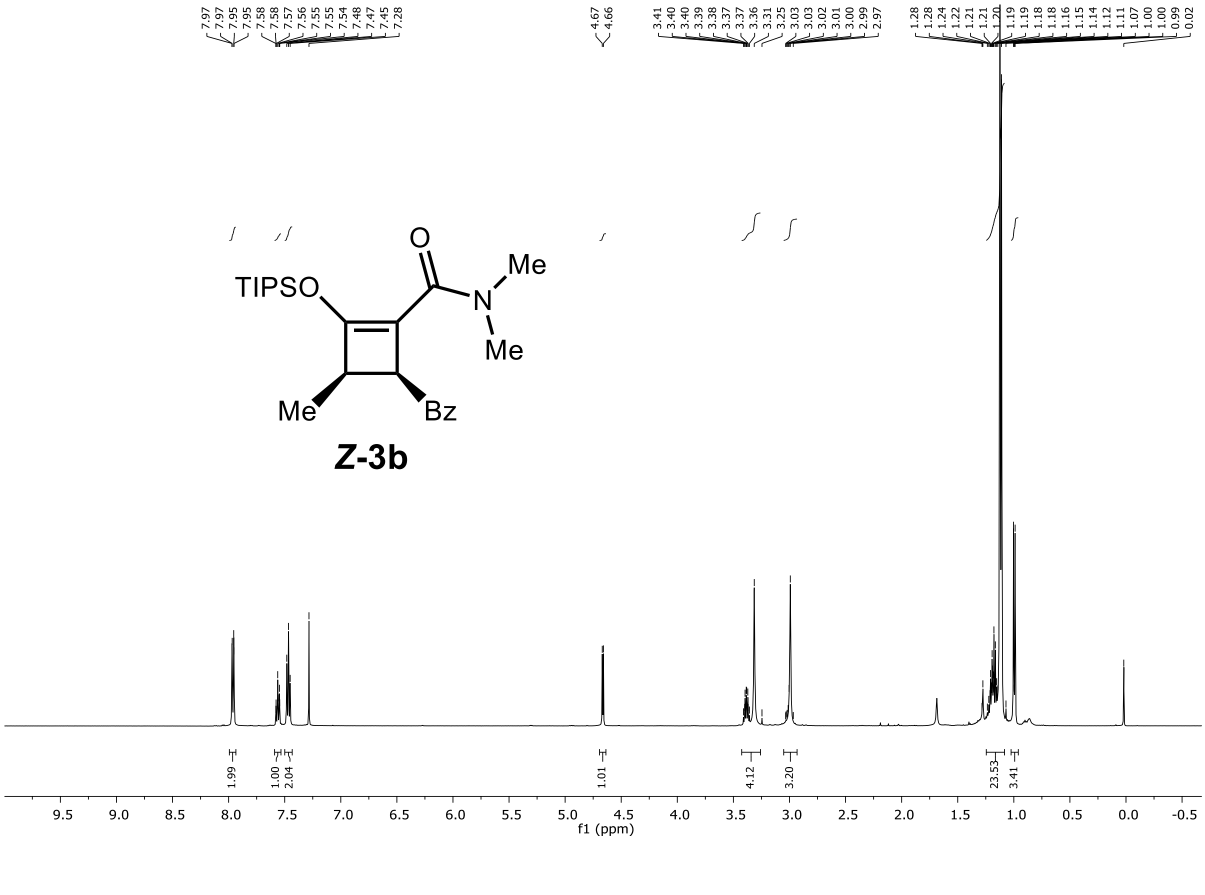
TIPSO
O
N
Me
Me
Me
Bz
Z-3b
f1 (ppm)

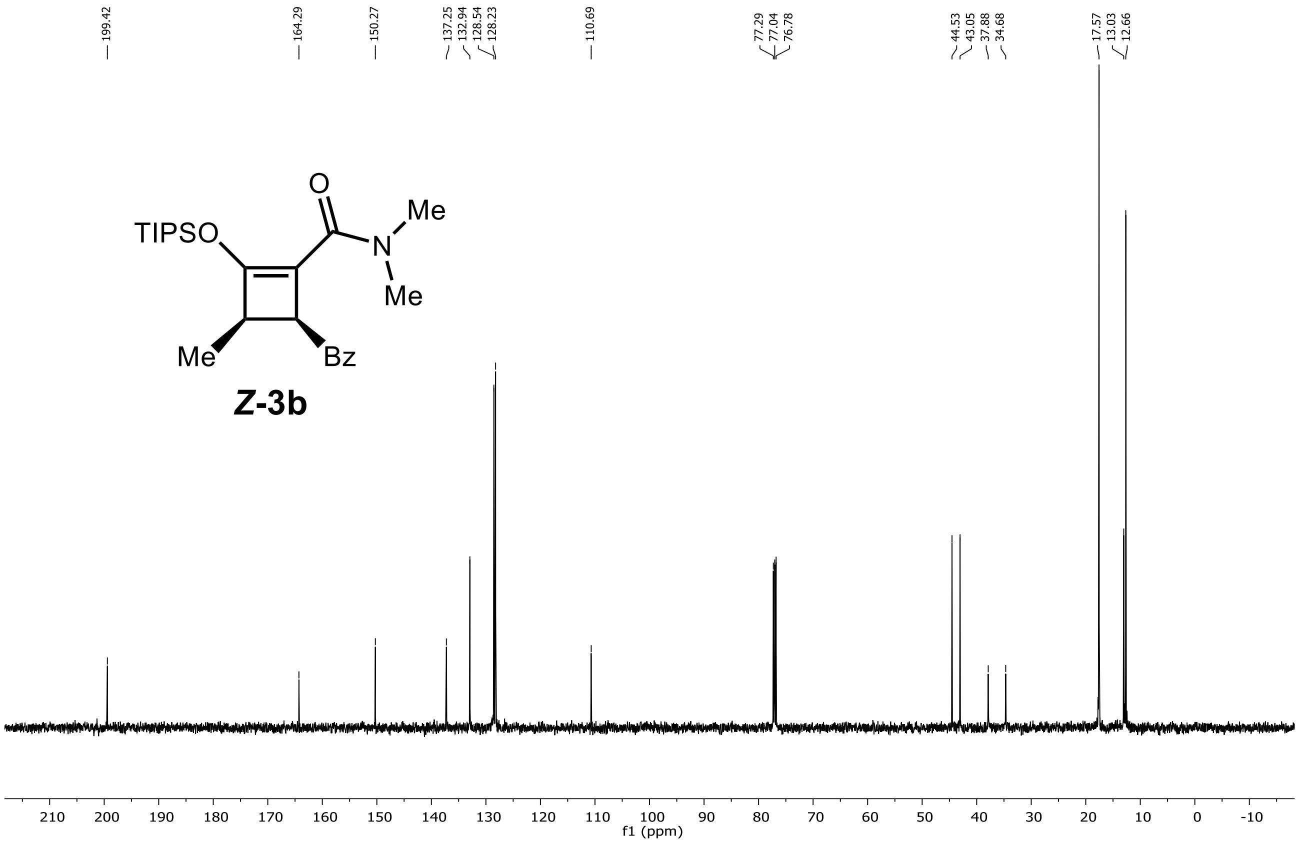
199.42
164.29
150.27
137.25
132.94
128.54
128.23
110.69
77.29
77.04
76.78
44.53
43.05
37.88
34.68
17.57
13.03
12.66
TIPSO
O
N
Me
Me
Me
Bz
Z-3b
210 200 190 180 170 160 150 140 130 120 110 100 90 80 70 60 50 40 30 20 10 0 -10
f1 (ppm)

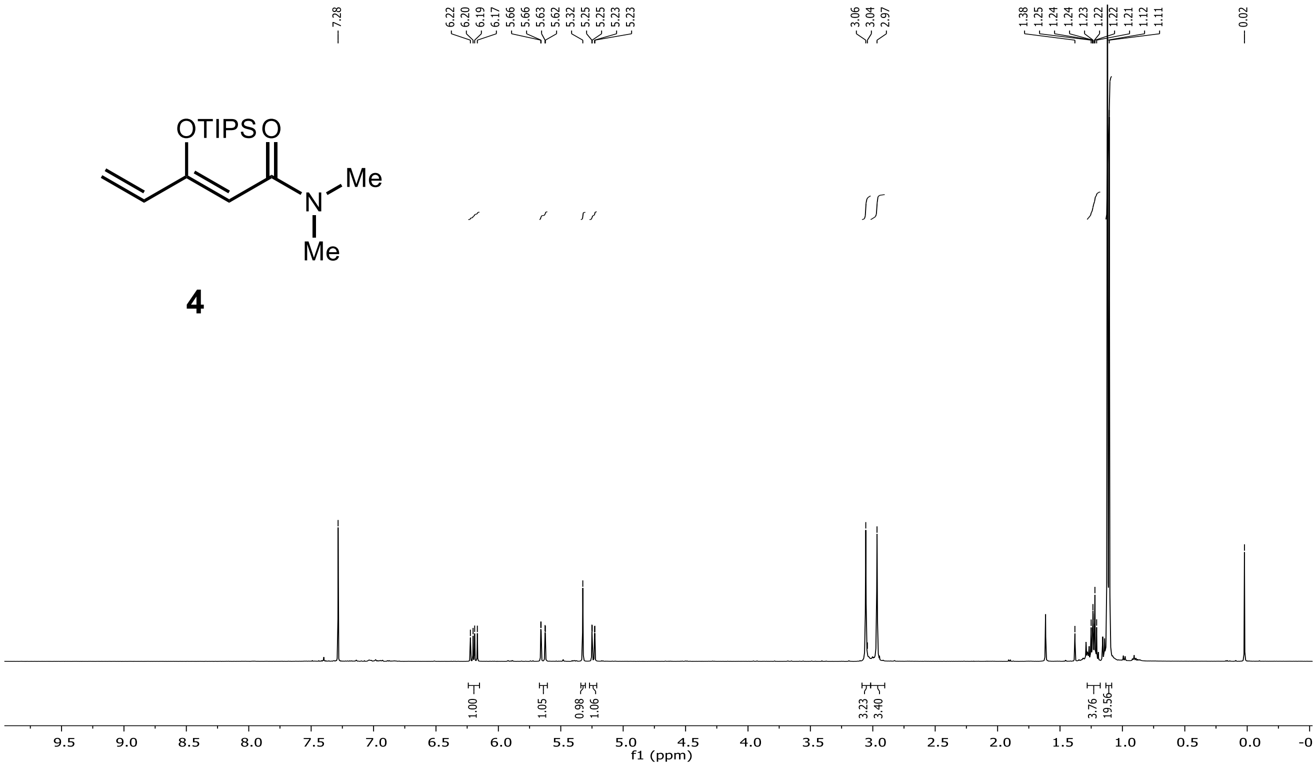
7.28
6.22
6.20
6.19
6.17
5.66
5.66
5.63
5.62
5.32
5.25
5.25
5.23
5.23
3.06
3.04
2.97
1.38
1.25
1.24
1.24
1.23
1.22
1.22
1.21
1.12
1.11
0.02
OTIPS
O
N
Me
Me
4
1.00
1.05
0.98
1.06
3.23
3.40
3.76
19.56
9.5 9.0 8.5 8.0 7.5 7.0 6.5 6.0 5.5 5.0 4.5 4.0 3.5 3.0 2.5 2.0 1.5 1.0 0.5 0.0 -0
f1 (ppm)

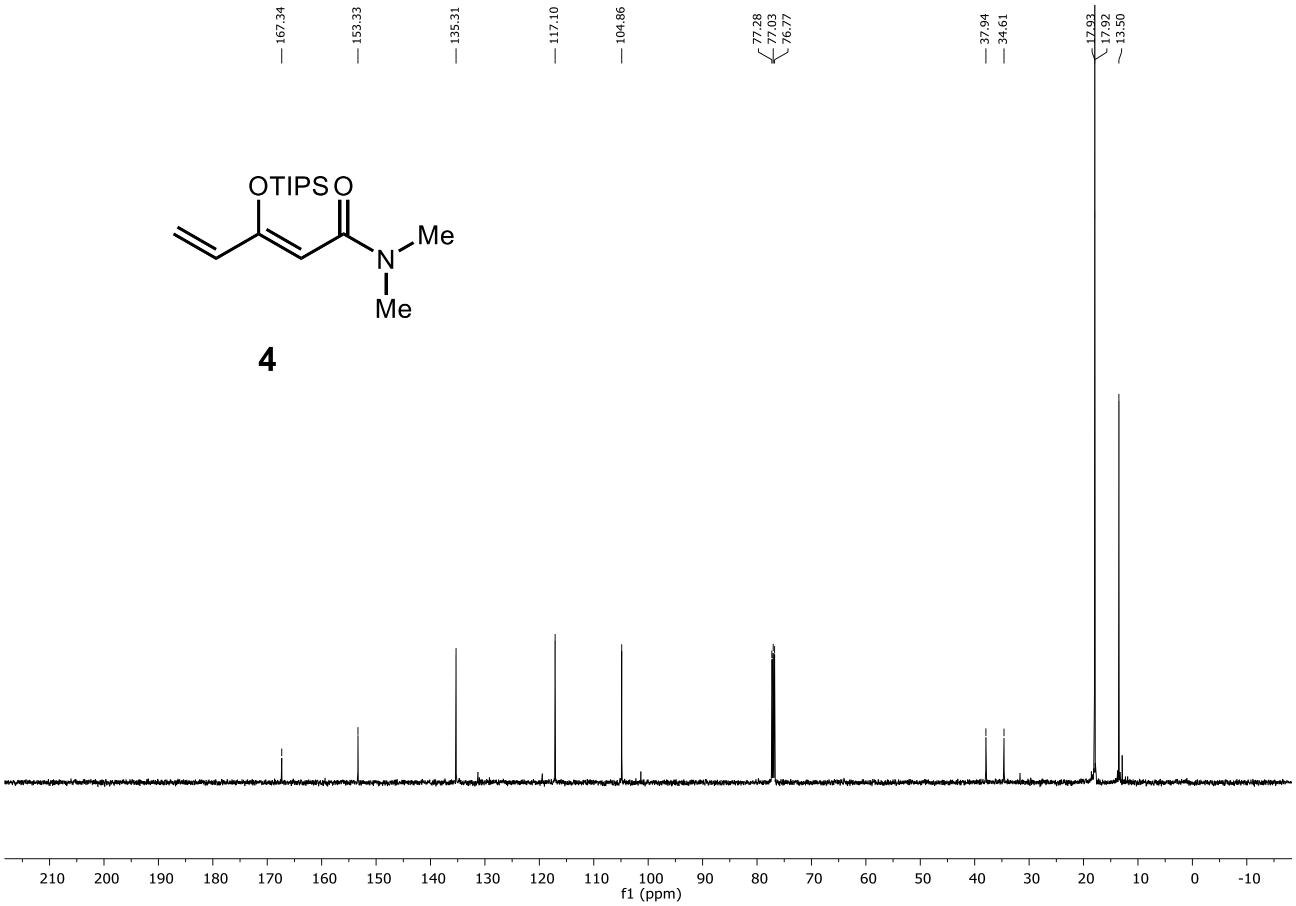

13. Selected HPLC Spectra:

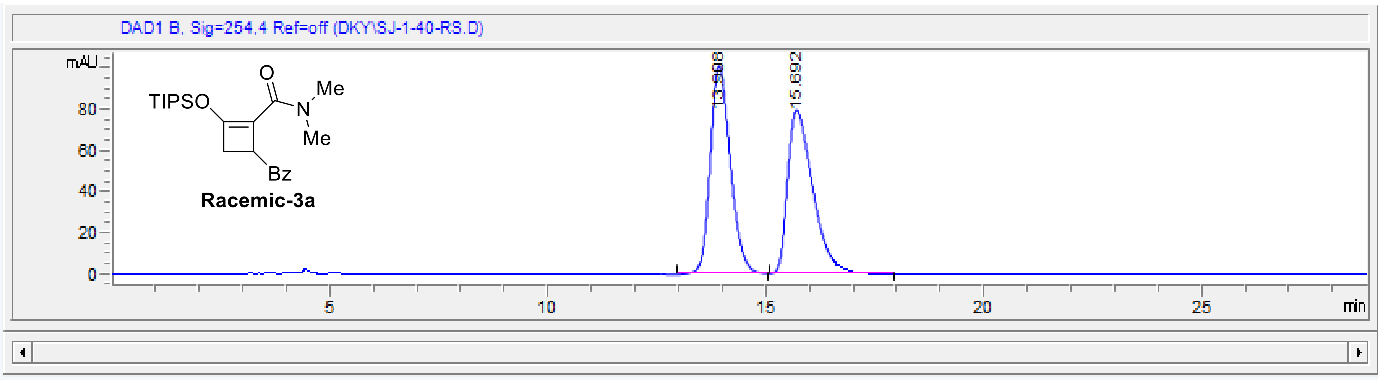

#	Time	Area	Height	Width	Area%	Symmetry
1	13.908	3303.2	102.3	0.4968	50.531	0.745
2	15.692	3233.8	80.5	0.6124	49.469	0.526

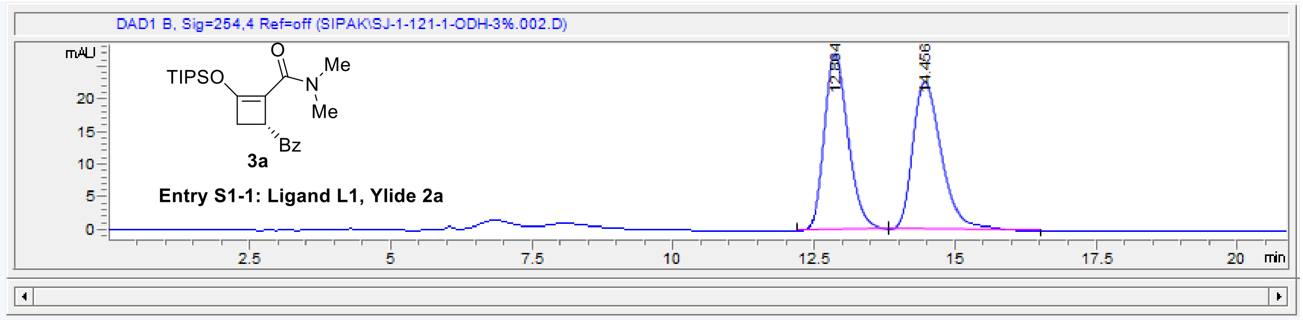

#	Time	Area	Height	Width	Area%	Symmetry
1	12.864	797.8	27	0.4547	49.902	0.776
2	14.456	801	22.6	0.5434	50.098	0.664

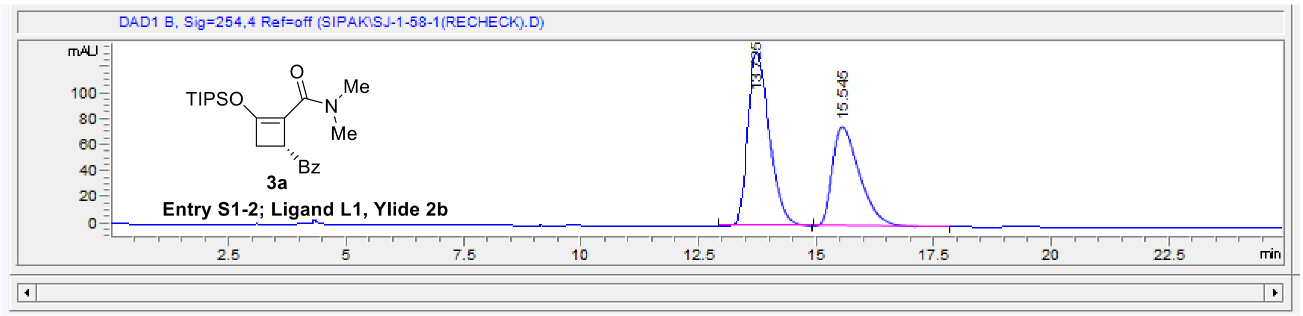

#	Time	Area	Height	Width	Area%	Symmetry
1	13.725	4297.7	133.5	0.4995	58.418	0.677
2	15.545	3059.1	75.7	0.6153	41.582	0.522

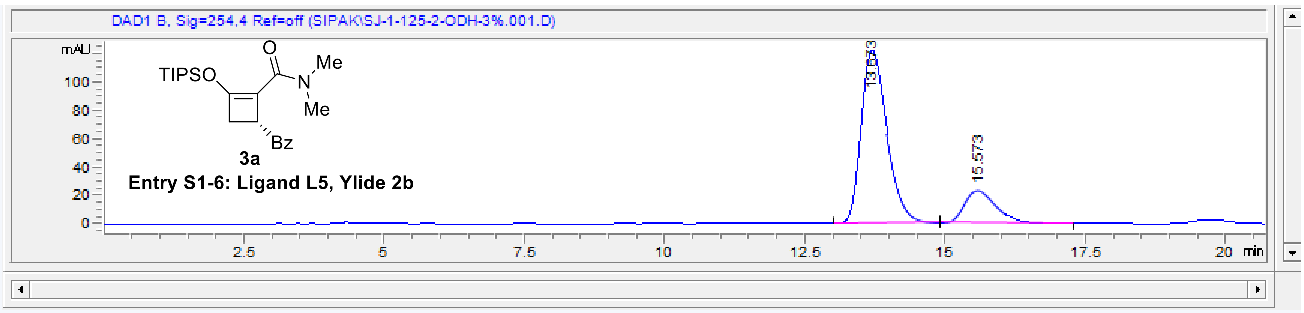

#	Time	Area	Height	Width	Area%	Symmetry
1	13.673	3983.2	122	0.5049	81.709	0.678
2	15.573	891.6	22.8	0.5996	18.291	0.655

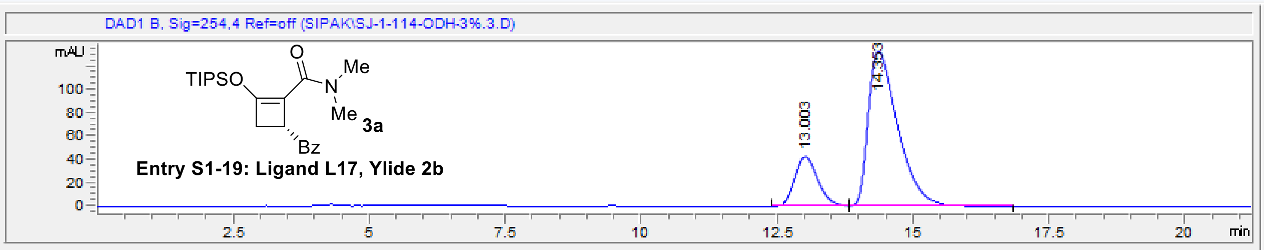

#	Time	Area	Height	Width	Area%	Symmetry
1	13.003	1266.4	42.5	0.46	20.087	0.765
2	14.353	5038.2	132.6	0.5771	79.913	0.489

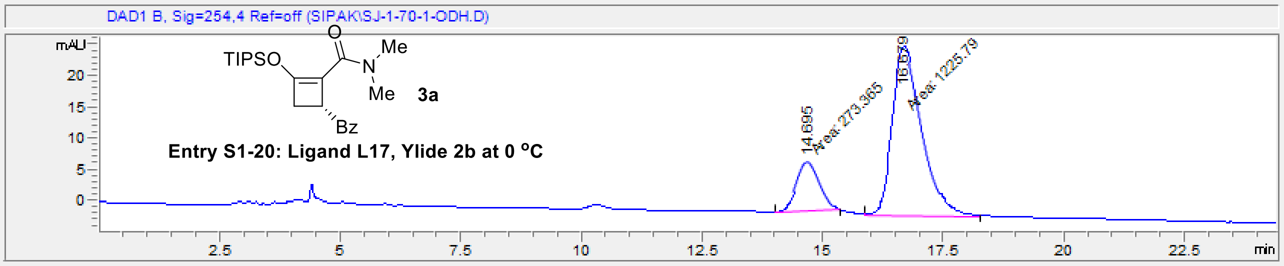

#	Time	Area	Height	Width	Area%	Symmetry
1	14.695	273.4	7.9	0.5745	18.235	0.902
2	16.679	1225.8	27.3	0.7479	81.765	0.629

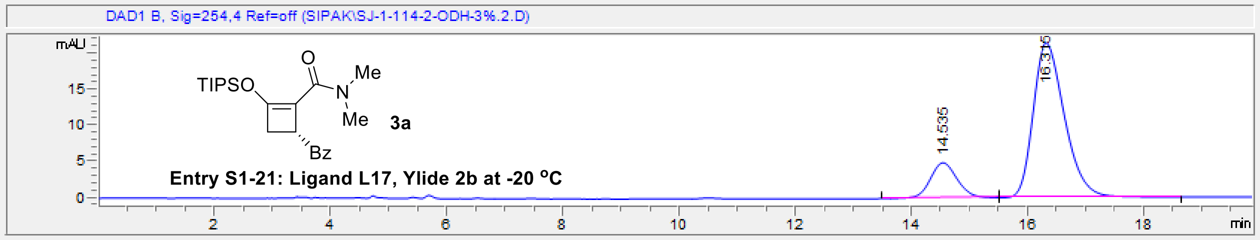

#	Time	Area	Height	Width	Area%	Symmetry
1	14.535	156.4	4.9	0.4895	16.313	0.874
2	16.315	802.2	21.3	0.58	83.687	0.684

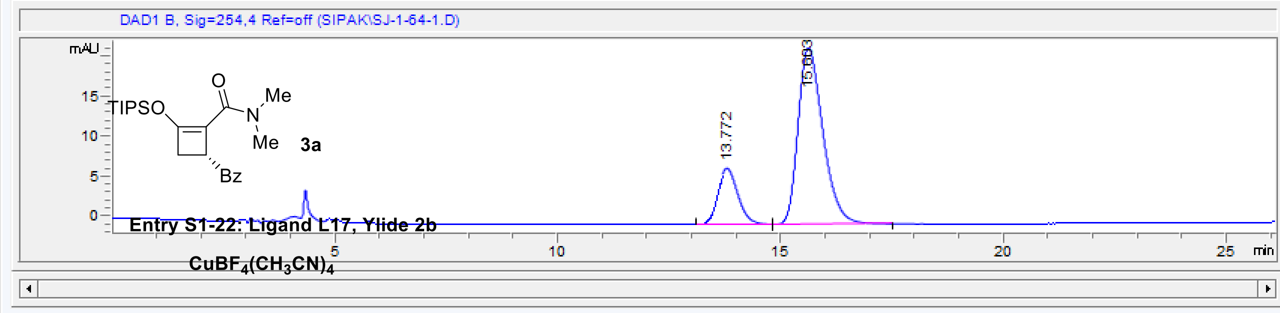

#	Time	Area	Height	Width	Area%	Symmetry
1	13.772	220.5	7	0.4709	20.657	0.828
2	15.603	846.9	21.8	0.5848	79.343	0.679

9. Selected HPLC Spectra of Cyclobutenecarboxamides **Z-3b** and **E-3b**

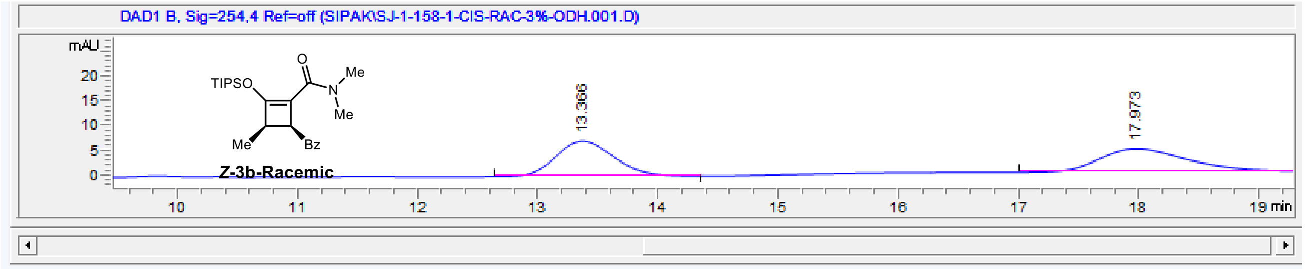

#	Time	Area	Height	Width	Area%	Symmetry
1	13.366	244.9	7.3	0.509	50.868	0.825
2	17.973	236.6	4.7	0.7163	49.132	0.692

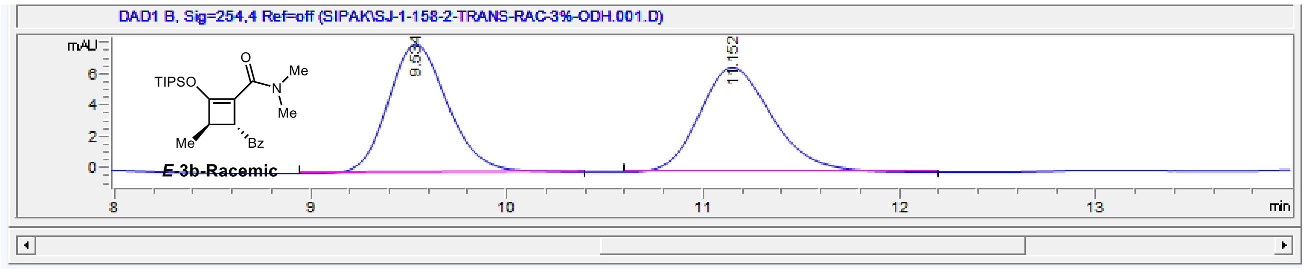

#	Time	Area	Height	Width	Area%	Symmetry
1	9.534	174.4	8.2	0.3284	50.308	0.819
2	11.152	172.3	6.6	0.3964	49.692	0.776

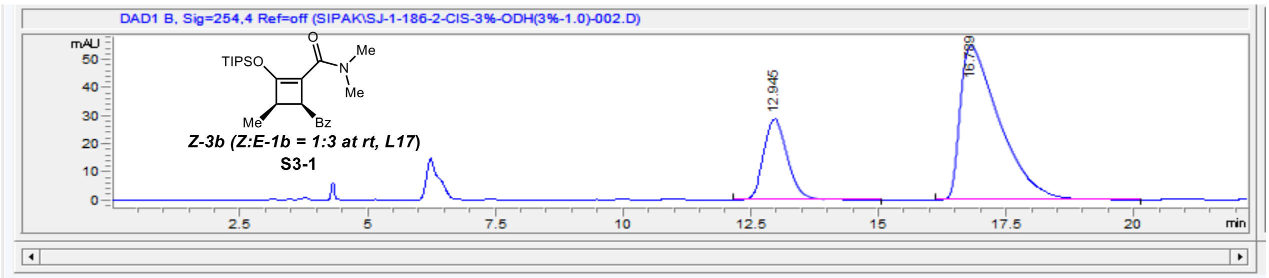

#	Time	Area	Height	Width	Area%	Symmetry
1	12.945	923	28.8	0.4963	23.733	0.769
2	16.789	2966.2	55.3	0.7938	76.267	0.35

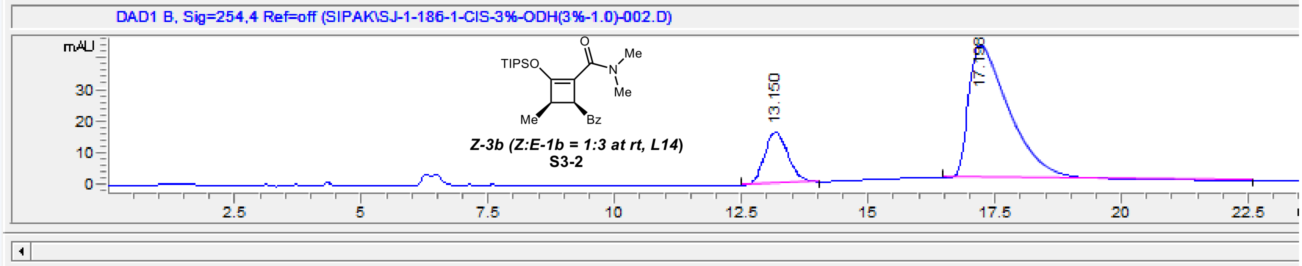

#	Time	Area	Height	Width	Area%	Symmetry
1	13.15	526.2	16.2	0.5035	18.263	0.82
2	17.198	2355.3	41.7	0.8248	81.737	0.374

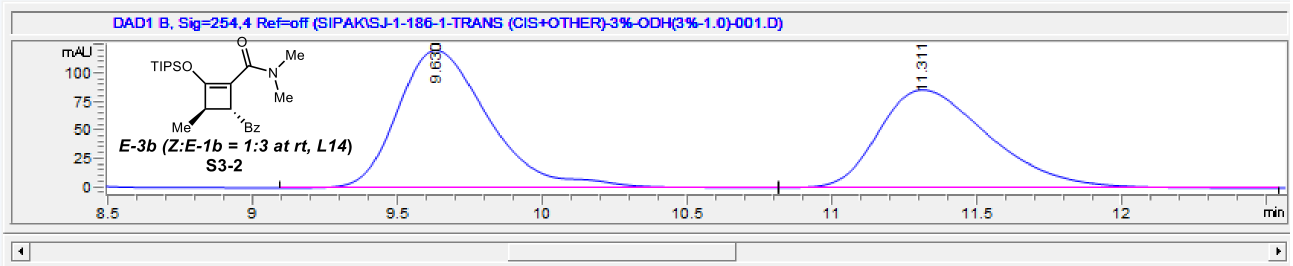

#	Time	Area	Height	Width	Area%	Symmetry
1	9.63	2630.3	120.1	0.3356	53.642	0.683
2	11.311	2273.2	85.5	0.4103	46.358	0.621

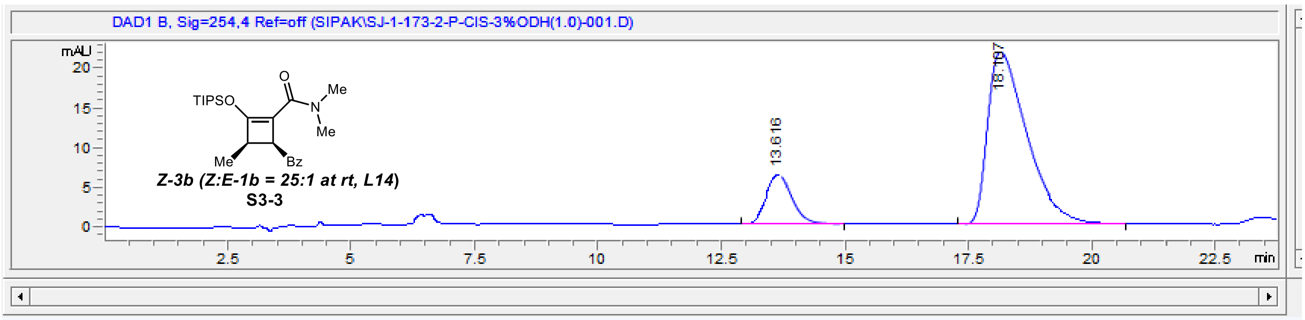

#	Time	Area	Height	Width	Area%	Symmetry
1	13.616	217.4	6.2	0.5287	15.334	0.805
2	18.107	1200.5	21.6	0.8051	84.666	0.462

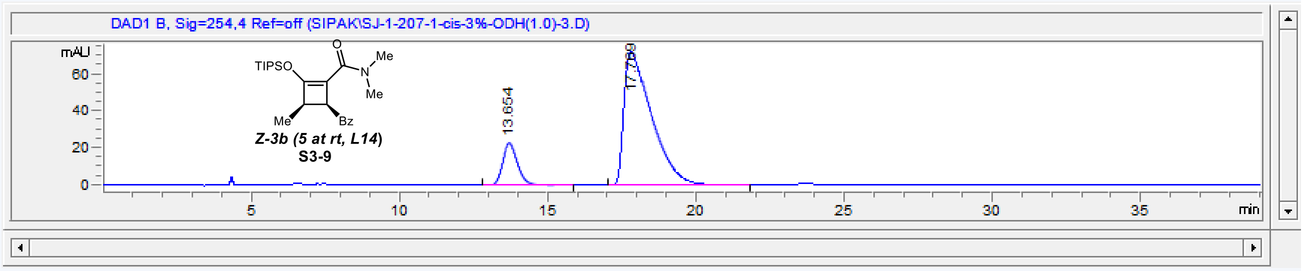

#	Time	Area	Height	Width	Area%	Symmetry
1	13.654	784.3	22.4	0.5377	14.544	0.783
2	17.769	4608	72.4	0.9268	85.456	0.323

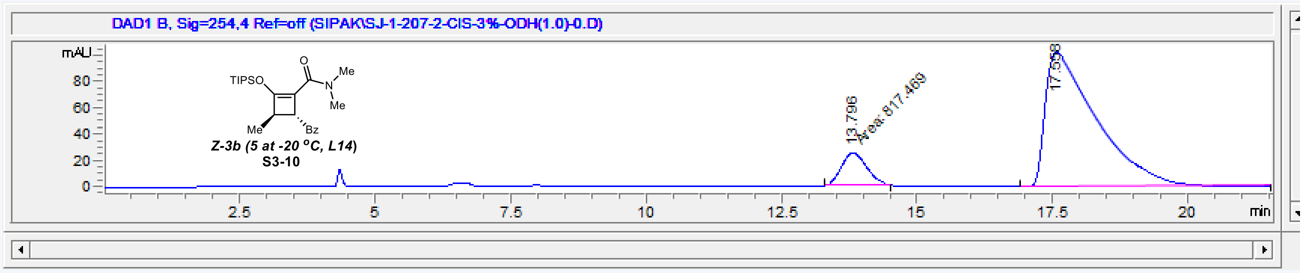

#	Time	Area	Height	Width	Area%	Symmetry
1	13.796	817.5	25.1	0.5429	10.573	0.811
2	17.558	6913.9	103	0.9475	89.427	0.259

(iv) HPLC result of E-3b (5 at -20 °C, L14):

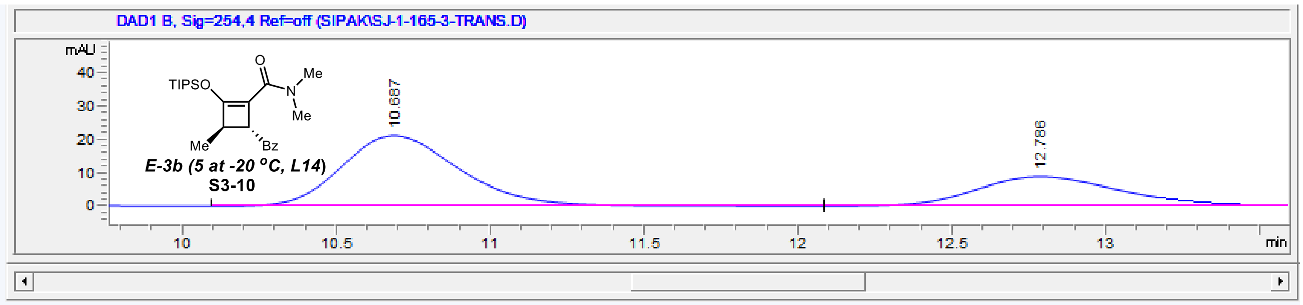

#	Time	Area	Height	Width	Area%	Symmetry
1	10.687	568.2	21.3	0.4116	65.769	0.756
2	12.786	295.7	8.9	0.509	34.231	0.75

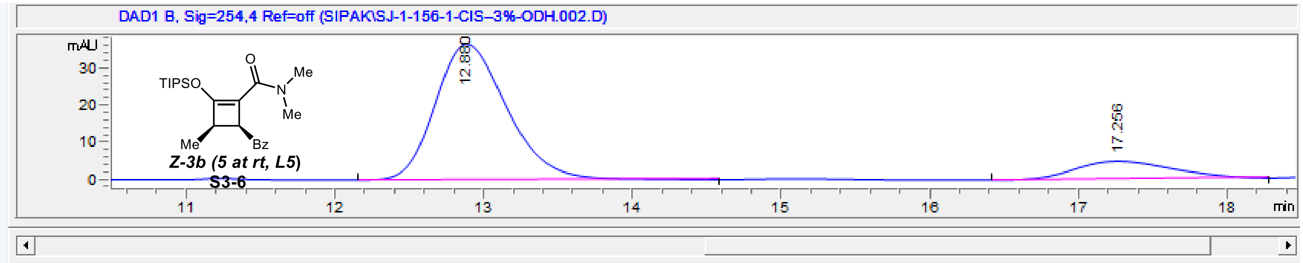

#	Time	Area	Height	Width	Area%	Symmetry
1	12.88	1223.3	36.5	0.5148	84.788	0.742
2	17.256	219.5	4.8	0.6835	15.212	0.755
